# PI3K/AKT signaling modulates transcriptional expression of EWS/FLI1 through specificity protein 1

**DOI:** 10.18632/oncotarget.5000

**Published:** 2015-08-22

**Authors:** Chiara Giorgi, Aleksandar Boro, Florian Rechfeld, Laura A. Lopez-Garcia, Maria E. Gierisch, Beat W. Schäfer, Felix K. Niggli

**Affiliations:** ^1^ Department of Oncology and Children's Research Center, University Children's Hospital, 8032 Zurich, Switzerland

**Keywords:** Ewing sarcoma, EWS/FLI1, PI3K pathway, promoter analysis

## Abstract

Ewing sarcoma (ES) is the second most frequent bone cancer in childhood and is characterized by the presence of the balanced translocation t(11;22)(q24;q12) in more than 85% of cases, generating a dysregulated transcription factor EWS/FLI1. This fusion protein is an essential oncogenic component of ES development which is necessary for tumor cell maintenance and represents an attractive therapeutic target. To search for modulators of EWS/FLI1 activity we screened a library of 153 targeted compounds and identified inhibitors of the PI3K pathway to directly modulate EWS/FLI1 transcription. Surprisingly, treatment of four different ES cell lines with BEZ235 resulted in down regulation of EWS/FLI1 mRNA and protein by ∼50% with subsequent modulation of target gene expression. Analysis of the EWS/FLI1 promoter region (−2239/+67) using various deletion constructs identified two 14bp minimal elements as being important for EWS/FLI1 transcription. We identified SP1 as modulator of EWS/FLI1 gene expression and demonstrated direct binding to one of these regions in the EWS/FLI1 promoter by EMSA and ChIP experiments. These results provide the first insights on the transcriptional regulation of EWS/FLI1, an area that has not been investigated so far, and offer an additional molecular explanation for the known sensitivity of ES cell lines to PI3K inhibition.

## INTRODUCTION

Ewing sarcoma (ES) is the second most frequent bone cancer in childhood. Clinically, ES appears as very aggressive osteolytic tumor with early tendency for development of metastasis [1]. It belongs to the group of small-round-blue-cell tumors and is comprised of largely undifferentiated cells. The unique feature of this tumor is the presence of the balanced t(11;22)(q24;q12) translocation in more than 85% of cases [2]. This gene rearrangement results in expression of a chimaeric fusion protein where the RNA binding domain of EWS is exchanged by the DNA binding domain of the ETS transcription factor FLI1, thus generating an aberrant transcription factor EWS/FLI1 [3–6]. More than 18 less represented alternative translocations involving EWS and other ETS protein family members have been described since [7–12].

Extensive evidence supports the notion that EWS/FLI1 is an essential oncogenic component of ES development. Its oncogenic activity is thought to be mediated through inappropriate regulation of target genes that are crucial for the fully malignant phenotype [5, 6, 13–17]. So far it is not known which of this target gene(s) act as crucial oncogenic driver(s). Hence, the prevalent hypothesis states that EWS/FLI1 is the major genetic mutation that is necessary for development and maintenance [18–24] of the tumor although it might not be sufficient. Since EWS/FLI1 expression is restricted to tumor cells, it represents an ideal therapeutic target. However, it acts as transcription factor, which in most cases are considered undruggable because of lack of enzymatic activity and their direct pharmacological inhibition is still challenging. Indeed, EWS/FLI1 behaves as intrinsically disordered protein and so far cannot be directly targeted by small molecules in a classical sense.

Therapy of ES today lacks specificity, is ineffective against metastasis and bears the potential of serious side effects. In the last few decades there has been considerable progress in both diagnosis as well as treatment of localized disease. However, only 15% of patients with metastatic disease survive and therefore this patient group needs specific attention. To advance future therapies, one of the available options lies in a better understanding of the biology of the fusion protein. Considering the difficulties in finding direct small molecule inhibitors for transcription factors, our aim is to study and characterize the cellular processes affecting or being affected by the fusion protein and thus providing indirect targeting possibilities. To identify molecular pathway(s) that might contribute to the transcriptional activity and oncogenic properties of EWS/FLI1, we therefore adopted a screening approach previously described [25], and screened a small molecule library that includes a broad range of protein kinase inhibitors covering all major signaling pathways. This approach led to the identification of SP1 as a direct regulator of EWS/FLI1 transcription through activation via the IGF/PI3K/AKT pathway, which is known to play a role in Ewing sarcoma [26–32] and whose blockage affects cell growth and survival [33–37]. Hence, we identify a critical regulatory mechanism upstream of EWS/FLI1.

## RESULTS

### Screening a library of small molecule inhibitors identifies PI3K pathway as modulator of EWS/FLI1 expression

To identify molecular pathways that may contribute to transcriptional activity and oncogenic properties of EWS/FLI1, we used the previously established and validated strategy [25] to screen a library of small molecule inhibitors covering a wide variety of molecular pathways. The collection of 153 inhibitors (Supplementary Table S1) was screened for EWS/FLI1 target gene modulation as primary read out in A673 ES cells at a final concentration of 500 nM. EWS/FLI1 transcriptional activity was monitored by expression of the known target genes pleckstrin homology-like domain, family A, member 1-PHLDA1 [25], Nuclear Receptor Subfamily 0 Group B Member 1-NROB1 [20], NK2 homeobox 2-NKX2.2 [21] and for EWS/FLI1 itself. Expression of PHLDA1 is repressed by EWS/FLI1, in contrast to NROB1 and NKX2.2 which are activated. General cytotoxicity of the compounds was determined by WST-1 assay, a colorimetric assay based on the cleavage of a tetrazolium salt, to form formazan in viable cells. The final hit-list was based on significant (*p* < 0.05, unpaired two-tailed *t*-test) modulation of at least two out of three target genes compared to untreated controls in A673 cells. The top 16 inhibitory compounds obtained from the screen are shown in Table 1 and included inhibitors targeting several signaling pathways, both known and unknown to play a role in sarcomas. The most prominent among them is the phosphoinositide-3-kinase (PI3K) pathway, which was affected by three different compounds. Inhibition of this pathway provoked a significant modulation of EWS/FLI1 target genes and a strong inhibition of cell proliferation in A673. Hence, these experiments identified PI3K signaling to modulate expression of EWS/FLI1 target genes.

**Table 1:** Screening of a small library of targeted compounds identifies PI3K pathway inhibitors as modulators of EWS/FLI1

EWS/FLI1 target gene modulation in A673 cells*,**								
Compound^a^	Target	Company	EWS/FLI1^b^	PHLDA1	NROB1	NKX2.2	Nr. of sign. Target gene response**	Proliferation^c^
NVP-BEZ235	PI3K/mTOR inhibitor	Axon 1281	89	**232**	**65**	**40**	**3**	58
PIK 75	PI3K/p110 alpha inhibitor	Axon 1334	**8**	115	9	**5**	**2**	54
NPV-BAG956	PI3K/PDK1 inhibitor	Axon 1282	111	**176**	83	**68**	**2**	68
DBZ	Gamma Secretase inhibitor	Axon 1488	116	**183**	**85**	**68**	**3**	113
BZ	Gamma Secretase inhibitor	Axon 1487	102	**181**	88	**77**	**2**	115
Vorinostat	HDAC inhibitor	Cayman	**47**	**185**	**61**	**68**	**3**	124
Bosutinib (SKI 606)	BCR-ABL/SRC inhibitor	Axon 1407	92	**221**	**84**	**56**	**3**	121
Tacrolimus	Calcineurin inhibitor	Axxonra	62	**126**	**71**	**67**	**3**	106
YM155	Survivin inhibitor	Selleck 1130	**25**	120	**28**	**46**	**2**	22
LY 2157299	TGF beta inhibitor	Axon 1491	73	153	**76**	**68**	**2**	108
Velcade	Proteosome inhibitor	Cilag	77	119	**17**	**18**	**2**	45
ICG-001	CBP/Beta-Catenin inhibitor	Selleck 2662	61	109	**72**	**69**	**2**	100
GDC-0449	Hedgehog Pathway inhibitor	Selleck 1082	97	147	**86**	**63**	**2**	114
Tandutinib	FLT3 inhibitor	Axon 1415	61	**137**	105	**66**	**2**	103
TG101348	JAK2 inhibitor	Symansis	**59**	**128**	97	**77**	**2**	100
NU 1025	PARP inhibitor	Axon 1370	90	**130**	87	**81**	**2**	120

a Treatment: 500 nM of compound for 24 hrs

b Relative mRNA expression levels of EWS/FLI1 and its target gene in percentage compared to DMSO. Significant values are written in bold (*p* < 0.05, unpaired two-tailed student *t*-test).

c Cell proliferation measurement using WST-1 assay was performed in parallel. Values are shown in percentage of untreated control (=100%) and represent mean of 2–4 independent experiments performed in duplicate.

* In % of control (=100%)

** *p* < 0.05, unpaired two-tailed *t*-test, significant values written in bold.

**Figure 1:**
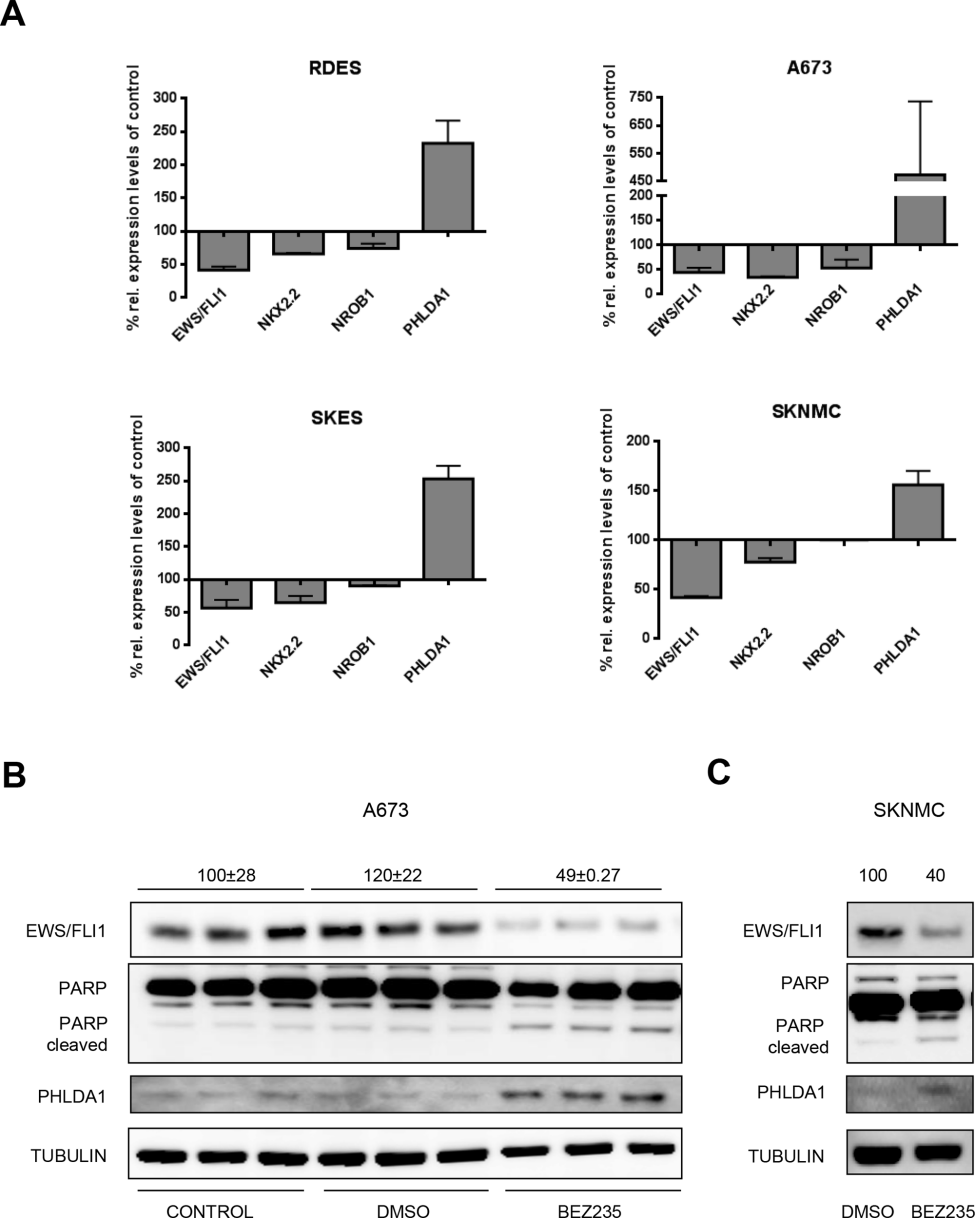
BEZ235 affects EWS/FLI1 levels **A.** Relative expression of EWS/FLI1 and its target genes measured by qRT-PCR after 24 hrs treatment with 500 nM BEZ235. Mean and standard deviation of 3 independent experiments. **B.** Protein level measured by western blot of EWS/FLI1, PHLDA1, PARP and TUBULIN as loading control in biological triplicates. Cells were treated for 24 h with 500 nM BEZ235 in A673 (B) and SKNMC cells **C.** (Numbers on top of the blot represent intensity of EWS/FLI1 bands measured by densitometry, indicated as mean compared to control and normalized to tubulin).

Among the PI3K inhibitors tested was BEZ235, which is a dual inhibitor of PI3K and the downstream mammalian target of Rapamycin (mTOR) that induced the most significant modulation of all three EWS/FLI1 target genes. Hence, we focused on this compound to further characterize modulation of EWS/FLI1 activity by the PI3K-mTOR pathway. Interestingly, upon treatment of four ES cell lines with 500 nM BEZ235 we observed a decrease of more than 50% of EWS/FLI1 mRNA levels itself (Figure 1A) that also resulted in a reduction of EWS/FLI1 protein levels (Figure 1B, 1C and Supplementary Figure S1) As expected, decrease of EWS/FLI1 mRNA led to modulation of target gene expression as well (NKX2.2, NROB1 and PHLDA1). Additional target genes such as insulin-like growth factor binding protein 3- IGFBP3 [19] and Lysyl Oxidase -LOX [38], repressed by EWS/FLI1, and six transmembrane epithelial antigen of the prostate 1-STEAP1 [39] and protein kinase C Beta -PRKCB [40], activated by EWS/FLI1, were found to be modulated as well (Supplementary Figure S2A–S2D).

Therefore, this data suggests that PI3K signaling is involved in transcriptional regulation of EWS/FLI1 expression.

### BEZ235 treatment induces cell cycle arrest

As described above, treatment with 500 nM BEZ235 for 24 hrs resulted in a decrease of EWS/FLI1 protein levels (Figure 1B and 1C) and as a consequence in PHLDA1 upregulation, which in turn led to a dose dependent reduction of viable cells compared to non-treated controls (Supplementary Figure S3A). To verify that the drug affected cell proliferation we stained the cells with crystal violet after drug treatment with 500 nM BEZ235 for 24 and 48 hrs. We observed a reduction of cell numbers by 40% and 70% compared to the DMSO control in A673 and 48% and 77% in SKNMC cells. Nevertheless, reduction in cell numbers was much more pronounced when cells were treated with Staurosporin or Nocodazole (Supplementary Figure S3B–S3E). Hence, BEZ235 seems to affect cell proliferation without decreasing viability. To investigate whether the compound induces cell death, we investigated PARP cleavage by Western blot. As shown in Figure 1B treatment with 500 nM BEZ235 resulted in minor PARP cleavage only. Subsequently, we investigated Casp3 and 7 activity both with the Casp3/7 Glo assay and at protein levels (Supplementary Figure S4A and S4B and data not shown). We observed no increase in activity of Casp3/7 after BEZ235 treatment, in contrast to treatment with Staurosporin and Nocodazole used as positive controls (increase by 5–6 fold). Hence, BEZ235 treatment did not induce apoptosis as measured by caspase activation and PARP cleavage. Subsequent cell cycle analysis after treatment with 500 nM BEZ235 for 24 and 48 hrs, both in A673 and SKNMC cells, revealed an increase in the cellular fraction in G1 phase. Indeed, the G1 population raised by 20% in A673 and 30% in SKNMC cells after drug treatment compared to DMSO control (Supplementary Figure S5A and S5B). Taken together, we conclude that BEZ235 treatment induces a cell cycle arrest, similar to what has been reported earlier [24]. Since the effect of BEZ235 on cell cycle progression could be due to inhibition of PI3K pathway or to EWS/FLI1 reduction, we also investigated the role of EWS/FLI1 in cell cycle progression. We depleted EWS/FLI1 both in A673 and SKNMC using 10 nM of siRNA for 48 hrs and analyzed the cell cycle distribution. Our results showed that depletion of EWS/FLI1 does not induce cell cycle arrest (Supplementary Figure S6A and S6B), but rather provokes a subG1 peak in SKNMC cells. This was also validated at protein level where EWS/FLI1 depletion provoked Casp7 activation and PARP cleavage in SKNMC (Supplementary Figure S6D), but not in A673 cells (Supplementary Figure S6C). In addition, crystal violet staining showed a reduction in cell numbers by 40% in SKNMC cells (Supplementary Figure S6F), but no effect on A673 cells (Supplementary Figure S6E). Taken together these data show that BEZ235 affects cell numbers mainly by inducing cell cycle arrest in ES cells.

### PI3K protein depletion reduces EWS/FLI1 expression

**Figure 2:**
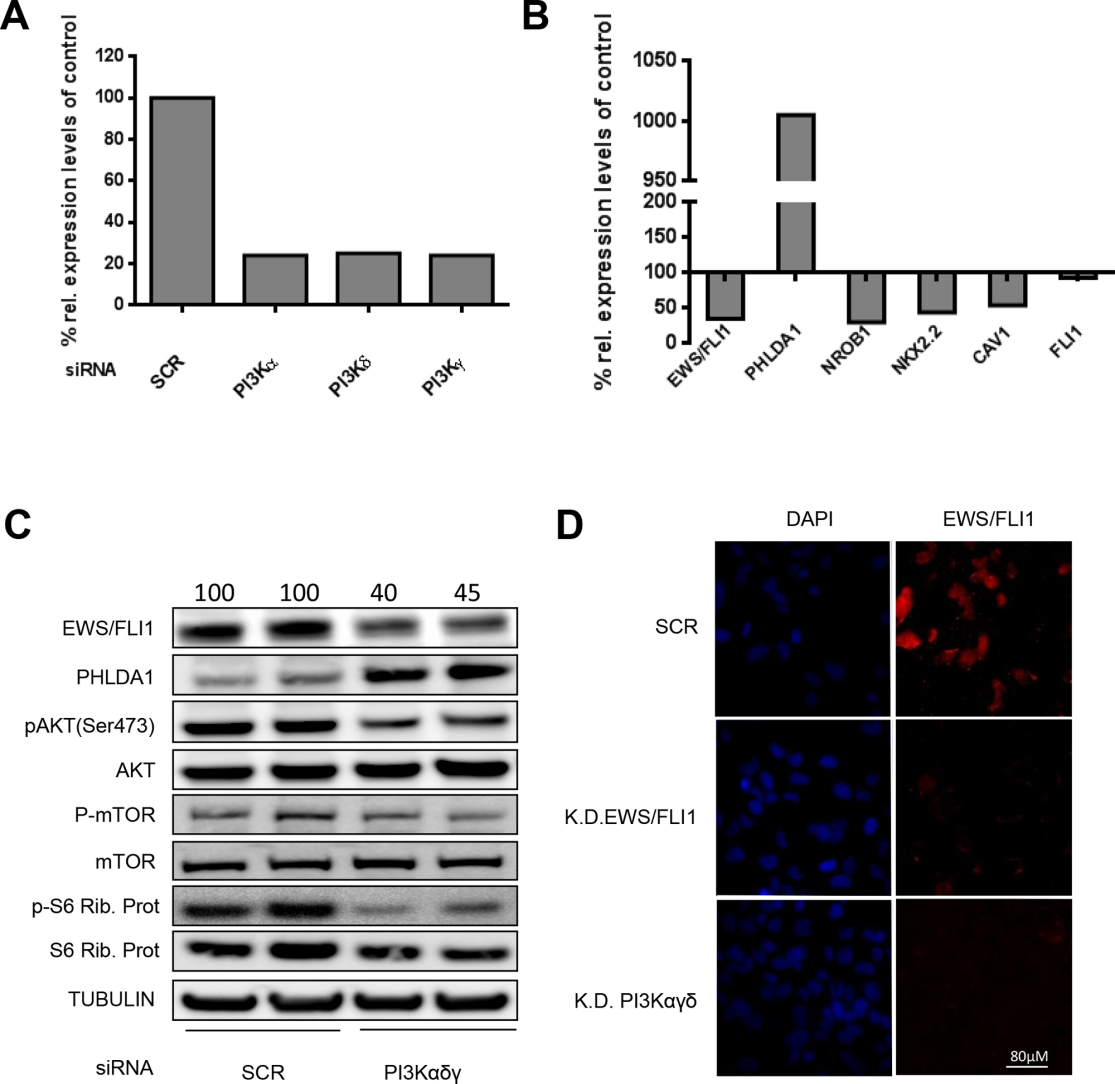
Modulation of EWS/FLI1 and target genes after PI3K pathway silencing **A.** PI3K α, γ, and δ mRNA expression levels were measured in A673 cells after silencing for 48 hrs compared to scrambled control by qRT-PCR. **B.** EWS/FLI1 and its target genes mRNA expression upon silencing of PI3K α, γ, and δ in A673 cells for 48 hrs. **C.** Expression levels of EWS/FLI1, PHLDA1 and PI3K downstream effectors after silencing in A673 cells for 48 hrs in biological duplicate (numbers on the top of the blot represent intensity of EWS/FLI1 bands measured by densitometry). **D.** Immunofluorescence assessment of FLI1 after silencing for 48 hrs of PI3Kαγδ; scrambled as control and FLI1 knock down as positive control. All the assays have been performed 3 times, shown are representative experiments.

To exclude off-target effects of the small molecule inhibitor BEZ235, we performed genetic loss-of-function experiments using siRNA targeting the catalytic domains α, δ and γ of class I PI3Ks in A673 and SKNMC cells. Silencing for 48 hrs resulted in down regulation of PI3K α, δ and γ mRNA by 75% compared to scrambled control as measured by quantitative RT-PCR (Figure 2A). In silenced cells PHLDA1 was up regulated by 10 fold, whereas target genes NROB1, NKX2.2 and Caveolin1 [41] were repressed by 70%, 55% and 45%, respectively (Figure 2B). Notably, expression of EWS/FLI1 itself was inhibited by 65%, whereas only a non-significant alteration of wt FLI1, used as negative control, was observed. These results were confirmed at the protein level since 48 hrs after treatment with PI3K specific siRNAs, EWS/FLI1 protein expression decreased while PHLDA1 protein level increased. As expected, silencing of PI3K decreased phosphorylation of its downstream effectors AKT, mTOR and S6 ribosomal protein as shown by phospho-specific antibodies (Figure 2C). The same result was confirmed in SKNMC cells where depletion of PI3Kαγδ led to a decrease of EWS/FLI1 protein levels, whereas the depletion of each single component did not affect the fusion protein (Supplementary Figure S7A). In addition, we performed immunofluorescence analysis to confirm the reduction of EWS/FLI1 after silencing of the PI3K components on the single cell level. As shown in Figure 2D (lower panel), after silencing of the three subunits, EWS/FLI1 is barely detectable anymore. Altogether, these results confirm regulation of EWS/FLI1 transcription by the PI3K pathway also at the genetic level. To validate that the effect on EWS/FLI1 target genes by the compound is due to the presence of EWS/FLI1, we performed the same assays in prostate cancer cells lacking the fusion protein. As shown in Supplementary Figure S7B, target genes of EWS/FLI1, while well expressed at endogenous levels in this cell type, are not affected by PI3K inhibition.

### PI3K signaling controls EWS/FLI1 transcription at its promoter

**Figure 3:**
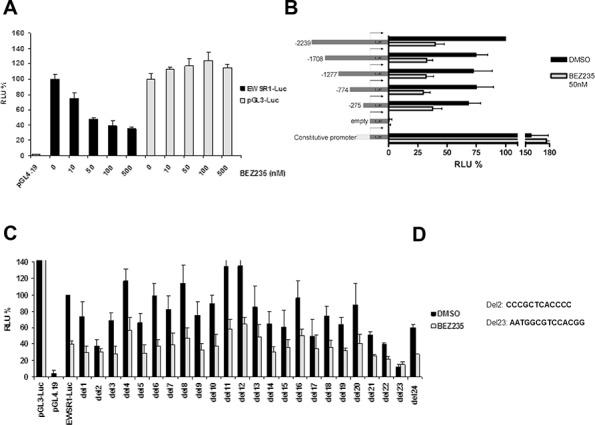
EWS/FLI1 promoter analysis by luciferase assay **A.** Relative luciferase activity in A673 cells transfected with different constructs (pGL4.19-empty vector control, pGL3- constitutive promoter control, EWSR1-Luc- 2.3kb promoter vector) upon treatment for 24 hrs with different concentration of BEZ235. **B.** Relative luciferase activity in A673 cells transfected with different deletion constructs of EWS/FLI1 promoter. Cells were treated with either DMSO or 50 nM BEZ235 **C.** Luciferase assay performed as above, with a series of deletion constructs of the 2.3kb EWS/FLI1 promoter **D.** Sequences of the two minimal binding elements that are absent from the deletion constructs Del2 and Del23 Mean and standard deviation of 3 independent experiments.

Since levels of wt FLI1 did not change upon inhibition of the PI3K pathway while at the same time EWS/FLI1 mRNA expression was reduced, we hypothesized that this control of EWS/FLI1 transcription occurs within the EWS promoter. To test this notion, we conducted reporter assays with a plasmid containing the 2.3 kb promoter of EWS in front of the luciferase gene. A673 ES cells were transfected with this construct and treated with increasing concentrations of BEZ235 for 24 hrs (Figure 3A). Interestingly, we observed a dose dependent decrease in luciferase activity which was not the case for a constitutive promoter thereby excluding effects of the compound on stability of luciferase itself. Already 50 nM of BEZ235 was able to reduce luciferase activity by 50% without affecting cell viability implying that indeed this promoter region contains a regulatory element responsive to PI3K signaling (Figure 3A). To narrow down the region of interest we designed several deletion constructs and performed reporter assays as before. Even the smallest construct of -275bp still responded to BEZ235 treatment. Hence, a regulatory element must be contained within this promoter element (Figure 3B).

To pinpoint further this potential regulatory element within the −275/+67 region of the promoter, we designed an additional series of deletion constructs in which 12–14bp were deleted in the context of the full size promoter (Figure 3C). If the responsible regulatory element is excised from the promoter we expect to lose any difference in luciferase activity upon drug treatment. Indeed, two constructs out of 24 did not respond to drug anymore, namely deletion 2 and deletion 23 (Figure 3D). Interestingly, both deletions also lost basal activity by more than 50% (Figure 3C). Therefore, our results suggest that inhibition of PI3K pathway affects gene expression of EWS/FLI1 mainly via two regions of the EWS promoter.

### SP1 is involved in transcriptional regulation of EWS/FLI1

**Figure 4:**
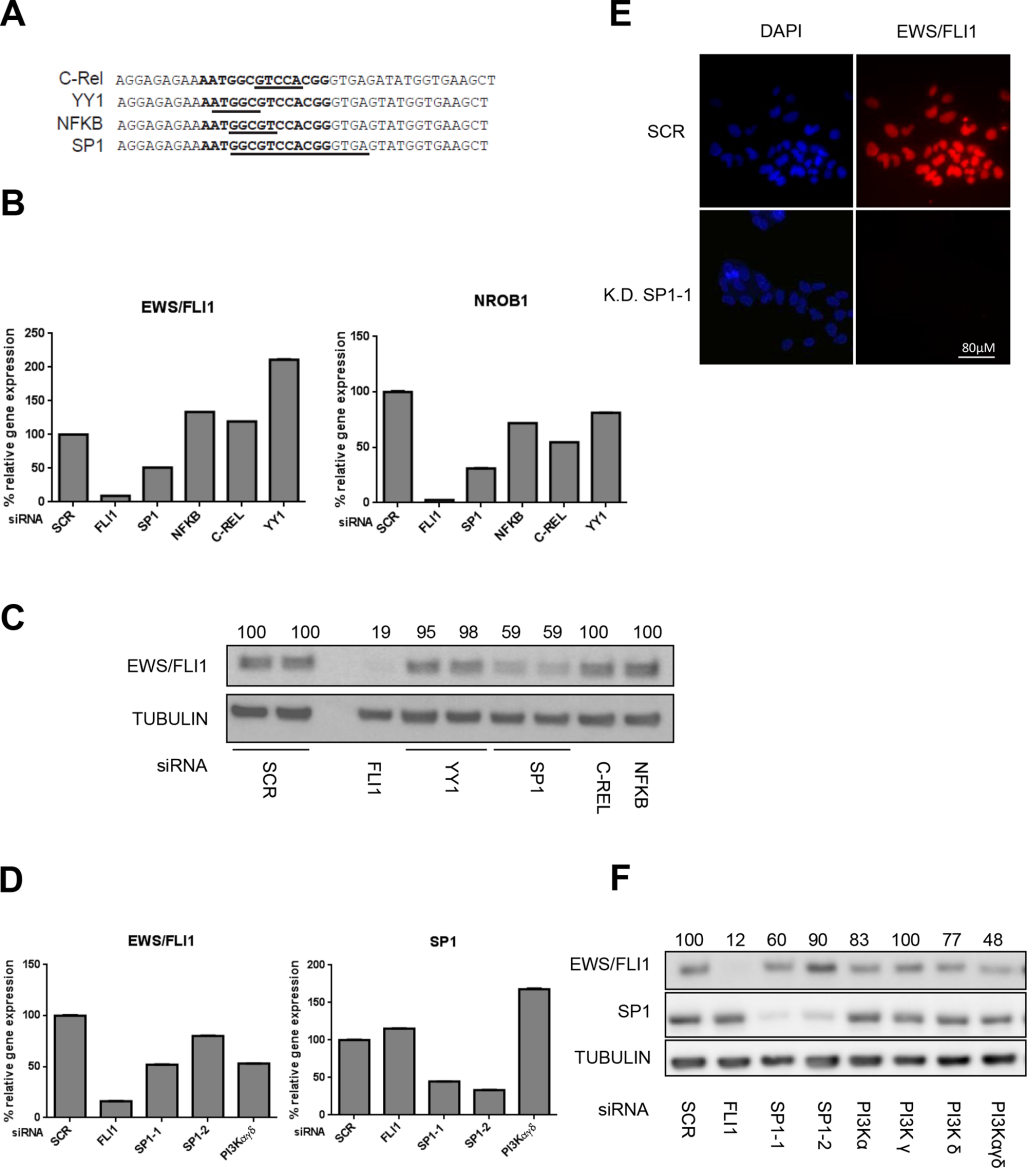
SP1 knock down affects EWS/FLI1 levels **A.** List of candidates which may bind the Del23 region of the promoter of EWS/FLI1 identified by Alibaba 2.2, Genome Browser and P-Match programs. **B.** siRNA mediated knock down for 48 hrs of candidate transcription factors to determine mRNA expression of EWS/FLI1 and target genes (via qRT-PCR). **C.** EWS/FLI1 protein level after silencing for 48 hrs of the candidate transcription factors by siRNA. **D.** siRNA mediated knockdown of PI3K components in order to determine mRNA expression of EWS/FLI1, and SP1 (via qRT-PCR) after 48 hrs. **E.** Immunofluorescence assessment of FLI1 after silencing of SP1 for 48 hrs. **F.** EWS/FLI1 protein level measured by western blot after 48 hrs reverse silencing of PI3K single subunits and combinations. Shown are representative experiments (*n* = 3).

**Figure 5:**
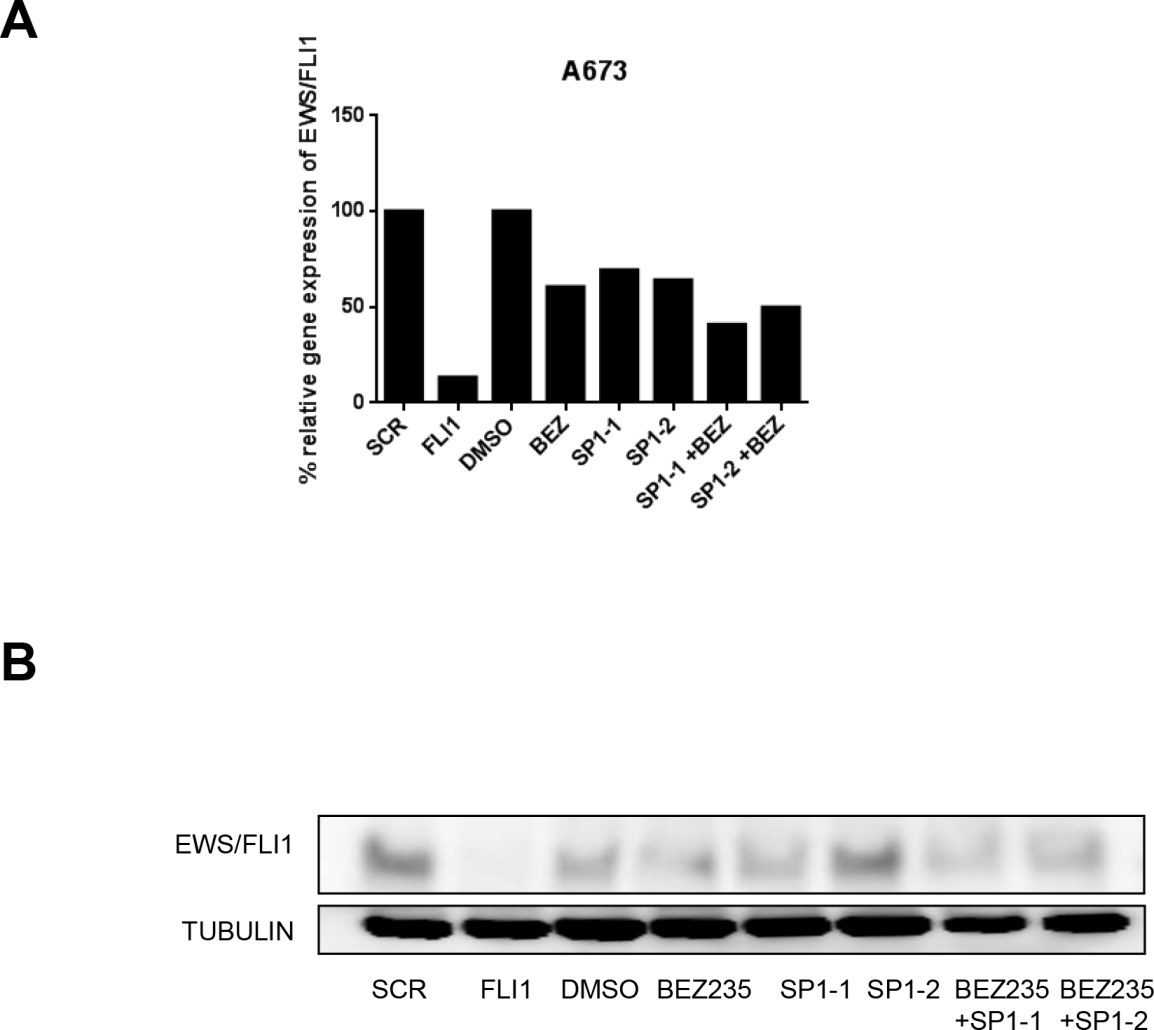
SP1 knock down in combination with BEZ235 treatment affects EWS/FLI1 levels EWS/FLI1 gene expression l **A.** and protein level **B.** after silencing of SP1–1 and SP1–2 by siRNA for 48 hrs or BEZ235 treatment for 24 hrs or the combination in A673 cells. Shown is a representative experiment (*n* = 3).

Our results point to an unknown transcription factor that binds to a specific region in the EWS promoter and whose activity can be stimulated by the PI3K pathway. Therefore, we used the sequence covered by Del23 to identify transcription factor candidates *in silico*. Using the programs Alibaba 2.2 and P-Match based on consensus sequences and Genome Browser based on ChIP datasets, we identified four potential candidates, C-Rel, YY1, NFKB and SP1, all known downstream targets of the PI3K pathway (Figure 4A). To determine which of these transcription factors might be involved in EWS/FLI1 gene expression, we performed siRNA depletion of each of the candidates and measured EWS/FLI1 gene expression together with its target gene NROB1 by qRT-PCR (Figure 4B) Knockdown of SP1 led to a reduction of EWS/FLI1 levels by 50% compared to control also at protein level (Figure 4C and Supplementary Figure S8A, S8B), whereas the other candidates did not affect EWS/FLI1 neither at the level of gene expression nor at protein level (Figure 4C and Supplementary Figure S9A–S9D). Also in this case, immunofluorescence analysis after SP1 depletion revealed barely detectable levels of EWS/FLI1, further strengthening the previous observation (Figure 4E and Supplementary Figure S10A and S10B). The same assays have been performed also in prostate cancer cells where SP1 depletion did not affect the levels of the target genes (Supplementary Figure S8C). To further validate our observations, we combined the knockdown of SP1 with 500 nM BEZ235 treatment for 24 hrs and observed an additive reduction of EWS/FLI1 levels (Figure 5A and 5B) both at gene expression and at protein level. Taken together we conclude that PI3K pathway regulates gene expression of EWS/FLI1 through SP1 activity.

**Figure 6:**
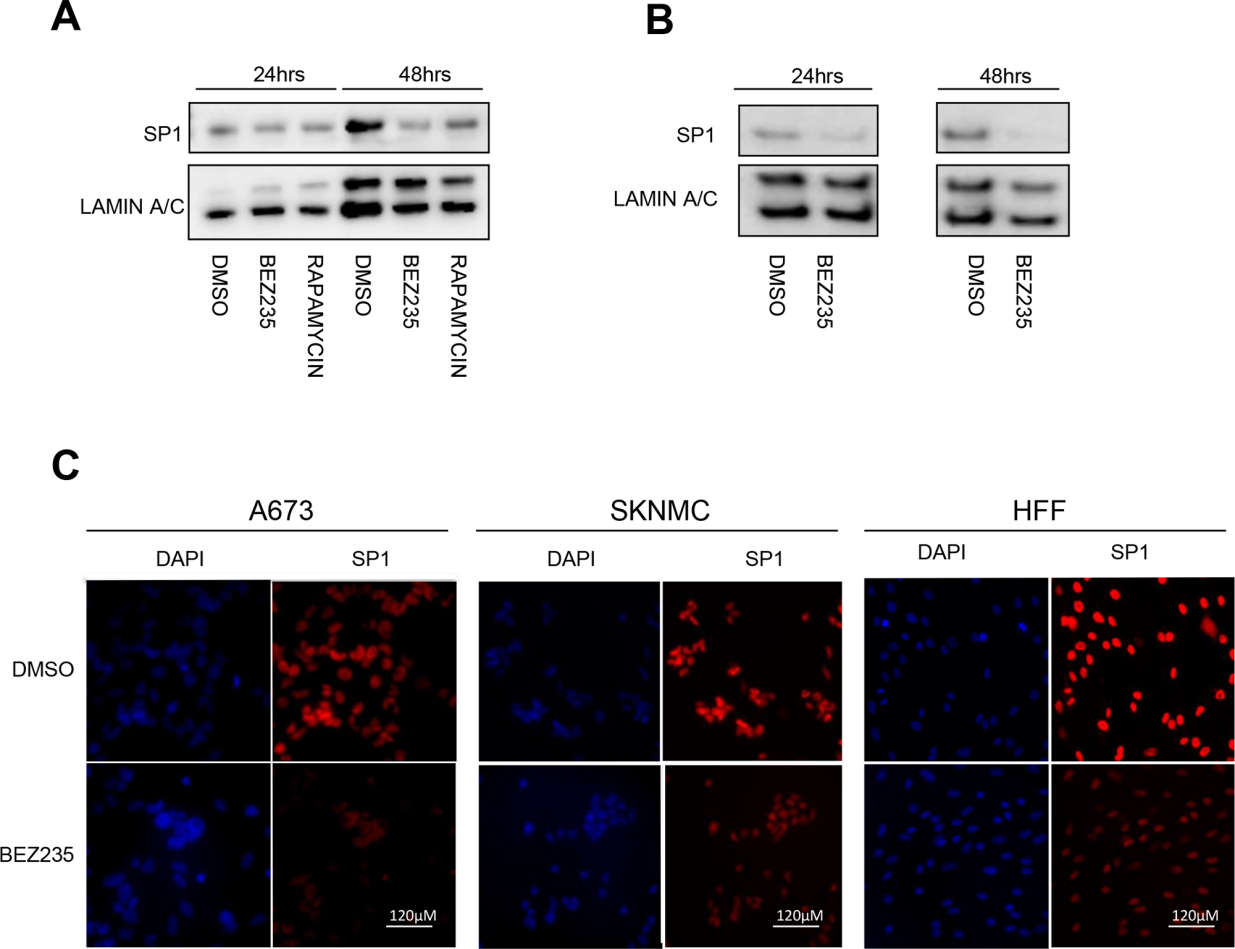
BEZ235 treatment affects SP1 levels Nuclear extracts of A673 and SKNMC cells were analyzed by western blot after BEZ235 and Rapamycin treatment for 24 and 48 hrs compared to DMSO control in A673 **A.** and in SKNMC using an SP1 specific antibody. **B.** Immunofluorescence assessment of SP1 after BEZ235 treatment for 24 hrs in A673 **C.** in SKNMC cells **D.** and in HFF **E.** Shown are representative experiments (*n* = 3).

To better understand the relation between the PI3K pathway and SP1 in ES cells, we investigated whether its activity is modulated by PI3K signaling. Since knock down of PI3K αγδ subunits induces a similar decrease of EWS/FLI1 mRNA and protein as depletion of SP1 (Figure 4D and 4F), we hypothesized that PI3K could directly affect SP1 levels. Since SP1 is a transcription factor and it is mainly located in the nucleus, we investigated SP1 protein levels after treatment for 24 and 48 hrs with either BEZ235 or Rapamycin, an inhibitor of the mTORC1 complex, in the nuclear fraction. Indeed, we observed a clear decrease of SP1 protein (Figure 6A and 6B). This was confirmed by immunofluorescence stainings showing a clear decrease of SP1 levels after 500 nM BEZ235 treatment also in a non-Ewing cell line such as human foreskin fibroblasts-HFF (Figure 6C). These results suggest that inhibition of PI3K pathway reduces SP1 activity, most likely via phosphorylation dependent mechanisms.

### SP1 directly binds to the Del23 region

**Figure 7:**
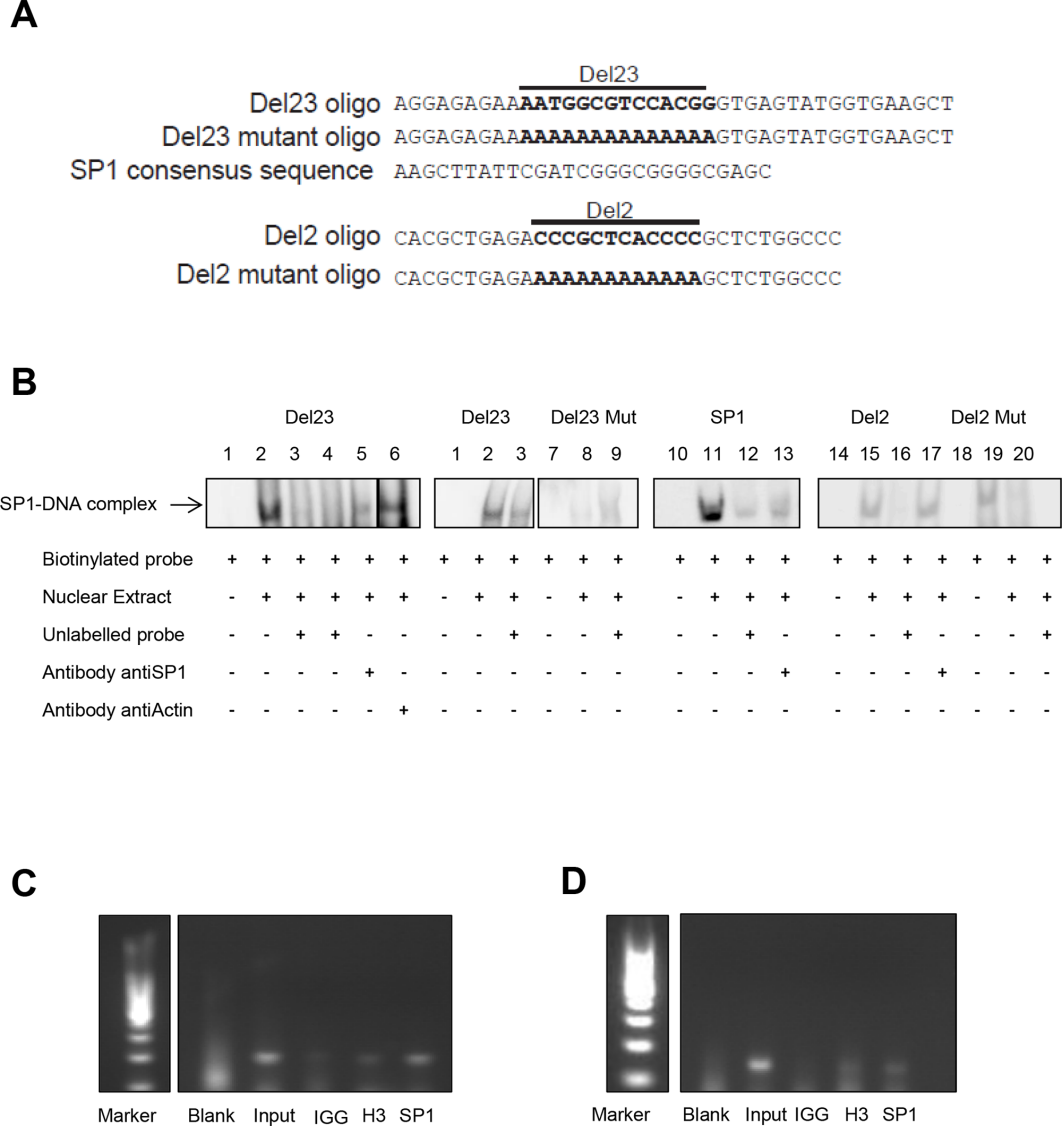
SP1 binds specifically to the Del23 region of the EWSR1 promoter **A.** Sequence of Del23, Del23 Mutant, Del2, Del2 Mutant and SP1 oligo. **B.** EMSA assay performed with nuclear extract of A673 cells. In lane 3 competitor oligonucleotide for Del23 was added; in lane 4 and 12 competitor oligonucleotide for SP1. In lane 9 we added Mutant Del23 oligonucleotide, in lane 16 one for Del2 and in lane 20 one for Del2 Mutant. In lane 5, 13 and 17 we added Sp1 antibody. In lane 6 we added Actin antibody. **C.** ChIP assay performed in A673 cells (C) and SKNMC cells **D.** Blank and IGG served as negative control, Input and H3, as positive ones. Shown are representative experiments (*n* = 3).

To demonstrate direct binding of SP1 to the Del23 region of the EWS/FLI1 promoter, we performed electrophoretic mobility shift assays using biotinylated double strand oligonucleotides covering the DNA sequence of Del23 (Figure 7A). Addition of nuclear extract produced a shift that could be competed by addition of an excess of unlabeled Del23 oligonucleotide (Figure 7B, lanes 2, 3), indicating that the Del23 region is indeed bound by protein. This shift could also be competed with an SP1 specific oligonucleotide (lane 4) as well as with a specific antibody against SP1 (lane 5), but not by addition of a control antibody (actin, lane 6). Specificity of the assay was further validated with a mutant Del23 oligonucleotide that generated a faint but unspecific shift (lane 8, 9) and with an SP1 specific oligonucleotide that could be displaced with the SP1 specific antibody (lane 13) similar to Del23. Since also Del2 has been implemented in EWS/FLI1 gene expression by the reporter assays and since also this region is GC rich, we tested Del2 in gel shift experiments as well. Del2 oligonucleotide produced a shift that could be competed with unlabeled oligo but not by addition of the SP1 antibody (lane 15, 17). Hence, these experiments suggest that the Del23 region of the EWS/FLI1 promoter is bound specifically by SP1 that does not bind to the Del2 sequence.

To verify this notion, we performed chromatin immunoprecipitation assays in two different Ewing cell lines (A673 and SKNMC) (Figure 7C and 7D). Using oligonucleotides spanning the Del23 region of the EWS promoter, a fragment could be enriched by immunoprecipitation with the SP1 antibody as well as with the control H3 antibody, but not with the unspecific IgG control. All together, these results indicate that SP1 indeed binds to the Del23 sequence in the promoter of EWS to modulate its transcriptional expression.

## DISCUSSION

Despite increasing efforts there are still no targeted agents implemented in routine therapy of Ewing sarcoma. Recently, several novel targeted approaches have been initiated and underwent clinical trials with limited success [42–45]. However, most of these efforts involved targeting of several enzymes downstream of the EWS/FLI1 fusion protein, such as IGF1R, and did not attempt to modulate the activity of this likely most crucial factor for ES oncogenesis itself.

Here, we conducted a screen of a library of small molecule targeted inhibitors affecting a broad range of different signaling pathways in order to define potential novel nodes directed at the fusion protein. For this, we employed the previously described and well established screening approach that uses expression of three EWS/FLI1 target genes, both repressed as well as activated, as surrogate markers of EWS/FLI1 activity [25]. As the most prominent pathway that was able to modulate EWS/FLI1 target gene expression the P3IK pathway emerged. This came as no surprise, since the importance of the IGFR1-PI3K-AKT axis has been demonstrated already in numerous studies [29, 30, 46–48], and triggered several clinical trials. However, using BEZ235, a dual PI3K/ mTOR inhibitor, as the most potent compound in our hit list we observed a strong decrease in EWS/FLI1 activity and surprisingly this occurs as consequence of a reduction at protein and RNA level of the fusion protein. In addition, since there was a reduction in cell number after treatment, also an effect on cell cycle progression has been noticed in agreement with Manara et al [34]. We hypothesize that the G1 arrest is due to the reduction of EWS/FLI1 levels, whereas further depletion causes cell death in SKNMC cells which are more sensitive than A673 (Supplementary Figure S6C). In agreement with the observed cell cycle arrest, reduced tumor growth was observed when mice were treated with BEZ235 after engraftment of TC71 ES cells [34], with regression induced when combined with vincristine.

Indeed, very little is known about the regulation of EWS/FLI1 transcription and even less about fusion protein turnover. So far, only one study was conducted that implied some possible regulatory regions in the EWSR1 promoter [49]. However, it has been shown previously that inhibition of mTOR by Rapamycin can decrease EWS/FLI1 protein levels [50] similar to what we observed with Rapamycin treatment in A673 cells (Figure 6A).

Inhibition of the PI3K pathway triggered repression of EWS/FLI1 transcription also when the pathway was genetically inhibited by specific siRNA treatment. However, we found that it was not sufficient to deplete one PI3K isoform but simultaneous down regulation of PI3K catalytic subunits α,γ and δ was necessary. This can probably be explained by the lack of mutations in any of these subunits in ES. Thus, the application of more isoform specific inhibitors might be limited and compensatory effects might explain the superior activity of BEZ235 from our panel of inhibitors tested.

Several additional inhibitors were identified from our screen, most notably two γ-secretase inhibitors. However, efforts to genetically verify a potential role of Notch receptors in EWS/FLI1 expression were not successful. This does not exclude a role of the pathway in ES biology as it has been demonstrate already that inhibition of notch can trigger neural differentiation of ES cells [51]. In addition, the most dramatic reduction in cell proliferation was seen with the survivin inhibitor YM155. Indeed, survivin protein and mRNA are found up regulated in ES cells, its expression constitutes a poor prognostic marker [52] and genetic knockdown reduced proliferation [53].

We used luciferase reporter assays to characterize for the first time a direct role of the PI3K-AKT-mTOR pathway in transcriptional regulation of the EWS/FLI1 promoter. This effect could be narrowed down to a regulatory element within the promoter, namely the Del23 region, that was bound by the transcription factor SP1 as shown by gel shift and ChIP assays. SP1 is ubiquitously expressed, and binds to GC rich motifs in general. Nevertheless, SP1 did not bind to additional GC-rich regions in the promoter such as Del2. SP1 has recently been described to be activated via phosphorylation by PI3K Cζ [54] and in addition, SP1 inhibitors, such as Mithramycin, have notable effect on EWS/FLI1 protein activity [55]. Mechanistically, it is still not entirely clear how SP1 activity is regulated by the PI3K pathway. However, we found that treatment specifically reduced SP1 levels, similar to what has been shown previously [56]. However, whether direct phosphorylation at one of the many known sites of SP1 is responsible for this effect, remains to be characterized.

Targeting IGFR1-PI3K-AKT-mTOR signaling has shown promising results in Ewing sarcoma [42, 44, 45, 57]. Our demonstration that inhibition of the pathway directly impairs expression of the fusion protein itself provides additional support for its therapeutic development [58]. Most promising appear to be combinations with other targeted agents that might modulate EWS/FLI1 activity such as YK-4–279 [59] or epigenetic modifiers that can potentially further suppress transcription of the fusion protein. Hence, elucidating the transcriptional regulation of EWS/FLI1 might provide additional molecular targets for this devastating disease.

## MATERIALs AND METHODS

### Cell lines

Three type 1 (A673, SKNMC, TC71) and two type 2 Ewing cell lines (SKES, RDES) were used. TC71 cells were kindly provided by Prof H. Kovar (St-Anna Children's Hospital, Vienna, Austria) and SKES and RDES by Prof. K.L. Schaefer (Institute of Pathology, Duesseldorf, Germany), A673, HFF and PC3 cells were purchased from the American Type Culture Collection-ATCC (Manassas, VA, USA). Cells were cultivated on 0.2% gelatin coated plates (Sigma-Aldrich, Buchs, Switzerland) in RPMI medium (DMEM for PC3 cells) supplemented with 10% FCS (Sigma-Aldrich), 1% Penicillin/Streptomycin (Thermo Fisher Scientific AG, Reinach, Switzerland), 1% L-glutamine (Bioconcept AG, Allschwil, Switzerland), at 37°C in 5% CO_2_.

### Screening assay

1.5 × 10^4^ cells were plated in 96-well plates 24 hrs prior to treatment. A library of 153 commercially available targeted inhibitors was acquired from AxonMedchem (Groningen, The Netherlands) and Selleck chemicals LLC (Munich, Germany) (See Supplementary Table S1). Compounds were added to cells in complete RPMI medium at a final concentration of 500 nM for 24 hrs. Lysis and subsequent cDNA synthesis was performed using AffinityScript QPCR cDNA Synthesis Kit (Agilent Technologies AG, Basel, Switzerland, #600559), followed by quantitative PCR (qPCR). Cell viability was measured in parallel using WST-1 cell proliferation kit (Roche Diagnostics AG, Rotkreuz, Switzerland).

### Quantitative PCR

Quantitative PCR (qPCR) was performed under universal cycling conditions on an ABI 7900 instrument using commercially available target probes and mastermix (all from Thermo Fisher Scientific AG). Data were analyzed using SDS 2.2 software (Thermo Fisher Scientific AG). CT values were normalized to glyceraldehyde-3-phosphate dehydrogenase (GAPDH). Relative expression levels of the target genes were calculated using the ΔΔCT method. All experiments were performed in triplicate and repeated independently at least 3 times. Data analysis was done with the GraphPad prism software (San Diego, CA, USA) and statistical analysis using the Student *t*-test. Commercially available target probes included (Thermo Fisher Scientific AG): EWSR1-FLI1:Hs03024807_ft, FLI1:Hs00956709_m1, SP1:Hs00916521_m1, PHLDA1:Hs00378285_g1, CAV 1: Hs00184697_m1, NKX2.2:Hs00159616_m1, NR0B1: Hs03043658_m1, GAPDH:Hs99999905_m1, PIK3α:Hs0 0907966_m1, PIK3 δ:Hs00192399_m1, PIK3 γ:Hs002770 90_m1, PRKCB:Hs00176998_m1, LOX:Hs00942480_m1, IGFBP3:Hs00365742_g1, STEAP1:Hs00185180_m1.

### siRNA treatments

A total of 2 × 10^6^ A673 cells were seeded per 60 mm dish. On the same day, transfection was carried out using LipofectamineRNAi MAX reagent (Thermo Fisher Scientific AG) and 10 nM siRNA of FLI1 (5266), SP1_1 (s13319), SP1_2 (s13320), C-Rel (s11906), NFKB (s9504), YY1 (s224779) PI3K α (s10520), PI3K δ (s10530), PI3K γ (s10532). As a negative control scrambled siRNA n^o^. 2 (s4390846) was used. All products were purchased from Thermo Fisher Scientific AG. Cells were lysed 48 hrs after silencing and subsequent RNA extraction using RNA easy mini kit (Qiagen Instruments AG, Hombrechtikon, Switzerland) was performed followed by cDNA synthesis with RT kit (Thermo Fisher Scientific AG).

### Immunoblotting

Cells were washed twice with PBS and harvested in lysis buffer containing 50 mM NaH_2_PO_4_ (pH 7.5), 150 mM NaCl, 1% Triton X-100, 1 mM Na3OV4, 5 mM Na-pyrophosphate, 40 nM NaF, 1 mM EGTA supplemented with protease inhibitor cocktail (Complete + 1 mM EDTA, Roche Diagnostics AG). For the preparation of nuclear fraction cells were lysed in 0.1% NP40, washed twice with PBS, centrifuged, and the supernatant discarded. The nuclear pellet was lysed in Roti-Load 1X (Carl Roth GmbH+Co. KG, Karlsruhe, Germany).

Protein concentration was determined by Bradford (Biorad, Reinach, Switzerland) and BCA (Thermo Fisher Scientific AG). 10–30 μg of protein extract was resolved on 4–12% SDS-PAGE and transferred onto nitrocellulose membrane (Thermo Fisher Scientific AG). Primary antibodies were used as follows: anti-FLI1 monoclonal antibody (MyBioSource LLC, San Diego, CA, USA, 1:1000), anti-PARP rabbit polyclonal antibody (Cell Signalling Technology, Berverly, MA, USA, 1:1000), anti-pAKT antibody (Ser473, Cell Signalling, 1:1000), anti-AKT antibody (Cell Signalling, 1:1000), anti-PHLDA1 antibody (Sigma Aldrich, 1:1000), anti-phospho-mTOR antibody (Ser2448, Cell Signalling, 1:1000), anti-mTOR antibody (Cell Signalling, 1:1000), anti-phospho-S6 Ribosomal protein antibody (Ser235/236, Cell Signalling, 1:2000), anti-S6 Ribosomal protein antibody (Cell Signalling, 1:1000), anti-SP1 antibody (Millipore, Billerica, MA, USA, 1:1000) and anti-β-tubulin I mouse monoclonal antibody (Sigma-Aldrich, dilution 1:40′000). After incubation with the appropriate secondary peroxidase-conjugated antibodies (1:1′000), detection was performed with the ECL chemiluminescence reagent (Amersham Biosciences, Freiburg, Germany).

### Luciferase assays

The promoter region of EWS/FLI1 (Ref. sequence NM_013986) covering 2.3kb (position -2239/+67 relative to the transcription initiation site) was cloned in pGL4.19 luciferase vector (Promega AG, Madison, WI, USA) using Infusion HD cloning kit (Clontech Laboratories, Inc Mountain View, CA, USA). With the same approach several deletion constructs of the EWS/FLI1 promoter were made, namely -1708/+67 -1277/+67, -774/+67, -275/+67. Using a site directed mutagenesis kit (Thermo Fisher Scientific AG), 24 additional deletion mutants of the -275/+67 construct were made (see Supplementary Table S2 for a detailed list of the plasmids). All constructs were verified by sequencing.

2 × 10^4^ A673 cells per well were plated in 96-well plate and transfected 24 hrs later using Jet Prime (Polyplus Transfection, Strasbourg, France) with 100 ng of reporter construct, or empty vector (pGL4.19) as a negative control. For normalization, cells were co-transfected with 10 ng of a renilla luciferase plasmid. After 24 hrs cells were treated with 50 nM BEZ235 or DMSO. 48 hrs post transfection they were lysed and assayed for luciferase activity using the Dual Glo luciferase reporter system (Promega AG).

### Electrophoretic mobility shift assays

1 × 10^7^ cells were plated, washed once with PBS 24 hrs later and lysed in Buffer A (10 mM Hepes pH 7.9, 10 mMKCl, 0.1 mM EDTA, 0.1 mM EGTA, 1 mM DTT, 0.5 mM PMSF) containing 10% NP40. The nuclear pellet was transferred to Buffer C (20 mM Hepes pH 7.9, 0.4 M NaCl, 1 mM DTT, 1x complete Mini-Roche tablet). 40 μg of nuclear extract, 100 nM of biotinylated oligo, 4 μM of unlabelled probe, 1 μL of anti-SP1 (Millipore) or anti Actin antibodies (Cell Signalling) were mixed. Oligonucleotides were ordered from Microsynth AG, Balgach, Switzerland) and annealed with Annealing Buffer (10 mM Tris, 1 mM EDTA, 50 mM NaCl pH 8.0):

Del23_Forward: AGGAGAGAA**AATGGCGTCC ACGG**GTGATATGGTGAAGCT (biotin);

Del23 mutant_Forward: AGGAGAGAA**AAAAA AAAAAAAAA**GTGAGTATGGTGAAGCT (biotin);

Del2_Forward: CACGCTGAGA**CCCGCTCACC CC**GCTCTGGCCC (biotin);

Del23 mutant_Forward: CACGCTGAGA**AAAAA AAAAAAA**GCTCTGGCCC (biotin);

SP1_Forward: AAGCTTATTCGATCGGGCGGGG CGAGC (biotin).

### ChIP assay

1 × 10^7^ cells were plated and cross-linked after 24 hrs with 1% formaldehyde (Thermo Fisher Scientific AG) for 5–10 minutes at RT. Formaldehyde was quenched by adding 125 mM Glycine (Sigma-Aldrich) for 5 minutes. Cells were collected, washed twice with cold PBS and lysed in IP buffer (150 mM NaCl, 50 mM Tris-HCl (pH 7.5), 5 mM EDTA, 0.5% NP-40, 1.0% Triton X-100). The crude extract was washed twice with IP buffer and sonicated 15 times for 20s [60]. Samples were incubated overnight at 4°C with the anti SP1 (Millipore), anti H3 (Cell Signalling) or anti IgG (Cell Signalling) antibodies and then immunoprecipitated (Active Motif kit). Primers (Microsynth AG) were as follows: CGAGTAAGCGGTGGTTCATC (forward).

### Immunofluorescence

Cells were washed once with PBS, fixed with 4% PFA, washed again with PBS and then with PBS-0.1% TritonX. Hereafter, they were incubated overnight with the primary antibody - FLI1 (1:50) (Sigma-Aldrich), SP1 (1:500) (Millipore), - diluted in PBS-0.1% TritonX with 4% Horse Serum (Sigma-Aldrich). Afterwards, cells were washed and incubated for 1 hr at room temperature with the secondary antibody (1:500) diluted in PBS - 4% horse serum (Sigma-Aldrich). Cells were washed twice with PBS and once with distilled water; one drop of Dapi (Vechtashield H-1200, Vector Laboratories, Inc. Burlingame, CA, USA) added and analyzed with a Zeiss inverted microscope.

### Casp3/7 assay

4 × 10^3^ A673 and SKNMC cells, were plated in a 384 well plate previously coated with 0.2% gelatin. After 24 hrs, cells were treated with 500 nM BEZ235, 1 μM Staurosporin, 100 nM Nocodazole or DMSO as controls. 24 hrs after treatment Caspase 3/7 reagent (Promega AG) was added in each well and luminescence was measured.

### FACS analysis

Treated cells were washed with PBS, collected, fixed with 70% Ethanol for 2 hrs on ice and stained with PI solution (20 μg/ml PI (Sigma-Aldrich), PBS- 0.1% TritonX 200 μg/ml RNAse A for measurement with a FACS Canto. Data were analyzed using Flow Jo program (Flow Jo LLC., Ashland, OR, USA).

## SUPPLEMENTARY FIGURES AND TABLES



## References

[1] Terrier P, Llombart-Bosch A, Contesso G (1996). Small round blue cell tumors in bone: prognostic factors correlated to Ewing's sarcoma and neuroectodermal tumors. Seminars in diagnostic pathology.

[2] Turc-Carel C, Aurias A, Mugneret F, Lizard S, Sidaner I, Volk C, Thiery JP, Olschwang S, Philip I, Berger MP (1988). Chromosomes in Ewing's sarcoma. I. An evaluation of 85 cases of remarkable consistency of t(11;22)(q24;q12). Cancer Genet Cytogenet.

[3] Delattre O, Zucman J, Plougastel B, Desmaze C, Melot T, Peter M, Kovar H, Joubert I, de Jong P, Rouleau G (1992). Gene fusion with an ETS DNA-binding domain caused by chromosome translocation in human tumours. Nature.

[4] May WA, Gishizky ML, Lessnick SL, Lunsford LB, Lewis BC, Delattre O, Zucman J, Thomas G, Denny CT (1993). Ewing sarcoma 11;22 translocation produces a chimeric transcription factor that requires the DNA-binding domain encoded by FLI1 for transformation. Proceedings of the National Academy of Sciences of the United States of America.

[5] May WA, Lessnick SL, Braun BS, Klemsz M, Lewis BC, Lunsford LB, Hromas R, Denny CT (1993). The Ewing's sarcoma EWS/FLI-1 fusion gene encodes a more potent transcriptional activator and is a more powerful transforming gene than FLI-1. Mol Cell Biol.

[6] Lessnick SL, Braun BS, Denny CT, May WA (1995). Multiple domains mediate transformation by the Ewing's sarcoma EWS/FLI-1 fusion gene. Oncogene.

[7] Jeon IS, Davis JN, Braun BS, Sublett JE, Roussel MF, Denny CT, Shapiro DN (1995). A variant Ewing's sarcoma translocation (7;22) fuses the EWS gene to the ETS gene ETV1. Oncogene.

[8] Kaneko Y, Yoshida K, Handa M, Toyoda Y, Nishihira H, Tanaka Y, Sasaki Y, Ishida S, Higashino F, Fujinaga K (1996). Fusion of an ETS-family gene, EIAF, to EWS by t(17;22)(q12;q12) chromosome translocation in an undifferentiated sarcoma of infancy. Genes Chromosomes Cancer.

[9] Ng TL, O'Sullivan MJ, Pallen CJ, Hayes M, Clarkson PW, Winstanley M, Sorensen PH, Nielsen TO, Horsman DE (2007). Ewing sarcoma with novel translocation t(2;16) producing an in-frame fusion of FUS and FEV. J Mol Diagn.

[10] Peter M, Couturier J, Pacquement H, Michon J, Thomas G, Magdelenat H, Delattre O (1997). A new member of the ETS family fused to EWS in Ewing tumors. Oncogene.

[11] Shing DC, McMullan DJ, Roberts P, Smith K, Chin SF, Nicholson J, Tillman RM, Ramani P, Cullinane C, Coleman N (2003). FUS/ERG gene fusions in Ewing's tumors. Cancer Res.

[12] Sorensen PH, Lessnick SL, Lopez-Terrada D, Liu XF, Triche TJ, Denny CT (1994). A second Ewing's sarcoma translocation, t(21;22), fuses the EWS gene to another ETS-family transcription factor, ERG. Nat Genet.

[13] Bailly RA, Bosselut R, Zucman J, Cormier F, Delattre O, Roussel M, Thomas G, Ghysdael J (1994). DNA-binding and transcriptional activation properties of the EWS-FLI-1 fusion protein resulting from the t(11;22) translocation in Ewing sarcoma. Mol Cell Biol.

[14] Hahm KB, Cho K, Lee C, Im YH, Chang J, Choi SG, Sorensen PH, Thiele CJ, Kim SJ (1999). Repression of the gene encoding the TGF-beta type II receptor is a major target of the EWS-FLI1 oncoprotein. Nat Genet.

[15] Nakatani F, Tanaka K, Sakimura R, Matsumoto Y, Matsunobu T, Li X, Hanada M, Okada T, Iwamoto Y (2003). Identification of p21WAF1/CIP1 as a direct target of EWS-Fli1 oncogenic fusion protein. J Biol Chem.

[16] Ohno T, Rao VN, Reddy ES (1993). EWS/Fli-1 chimeric protein is a transcriptional activator. Cancer Res.

[17] Kauer M, Ban J, Kofler R, Walker B, Davis S, Meltzer P, Kovar H (2009). A molecular function map of Ewing's sarcoma. PLoS One.

[18] Chansky HA, Barahmand-Pour F, Mei Q, Kahn-Farooqi W, Zielinska-Kwiatkowska A, Blackburn M, Chansky K, Conrad EU, Bruckner JD, Greenlee TK, Yang L (2004). Targeting of EWS/FLI-1 by RNA interference attenuates the tumor phenotype of Ewing's sarcoma cells *in vitro*. Journal of orthopaedic research : official publication of the Orthopaedic Research Society.

[19] Prieur A, Tirode F, Cohen P, Delattre O (2004). EWS/FLI-1 silencing and gene profiling of Ewing cells reveal downstream oncogenic pathways and a crucial role for repression of insulin-like growth factor binding protein 3. Mol Cell Biol.

[20] Kinsey M, Smith R, Lessnick SL (2006). NR0B1 is required for the oncogenic phenotype mediated by EWS/FLI in Ewing's sarcoma. Molecular cancer research : MCR.

[21] Smith R, Owen LA, Trem DJ, Wong JS, Whangbo JS, Golub TR, Lessnick SL (2006). Expression profiling of EWS/FLI identifies NKX2.2 as a critical target gene in Ewing's sarcoma. Cancer cell.

[22] Stegmaier K, Wong JS, Ross KN, Chow KT, Peck D, Wright RD, Lessnick SL, Kung AL, Golub TR (2007). Signature-based small molecule screening identifies cytosine arabinoside as an EWS/FLI modulator in Ewing sarcoma. PLoS medicine.

[23] Owen LA, Lessnick SL (2006). Identification of target genes in their native cellular context: an analysis of EWS/FLI in Ewing's sarcoma. Cell cycle.

[24] Crompton BD, Stewart C, Taylor-Weiner A, Alexe G, Kurek KC, Calicchio ML, Kiezun A, Carter SL, Shukla SA, Mehta SS, Thorner AR, de Torres C, Lavarino C, Sunol M, McKenna A, Sivachenko A (2014). The genomic landscape of pediatric Ewing sarcoma. Cancer Discov.

[25] Boro A, Pretre K, Rechfeld F, Thalhammer V, Oesch S, Wachtel M, Schafer BW, Niggli FK (2012). Small-molecule screen identifies modulators of EWS/FLI1 target gene expression and cell survival in Ewing's sarcoma. Int J Cancer.

[26] Yee D, Favoni RE, Lebovic GS, Lombana F, Powell DR, Reynolds CP, Rosen N (1990). Insulin-like growth factor I expression by tumors of neuroectodermal origin with the t(11;22) chromosomal translocation. A potential autocrine growth factor. The Journal of clinical investigation.

[27] Hamilton G, Mallinger R, Hofbauer S, Havel M (1991). The monoclonal HBA-71 antibody modulates proliferation of thymocytes and Ewing's sarcoma cells by interfering with the action of insulin-like growth factor I. Thymus.

[28] van Valen F, Winkelmann W, Jurgens H (1992). Type I and type II insulin-like growth factor receptors and their function in human Ewing's sarcoma cells. Journal of cancer research and clinical oncology.

[29] Scotlandi K, Benini S, Sarti M, Serra M, Lollini PL, Maurici D, Picci P, Manara MC, Baldini N (1996). Insulin-like growth factor I receptor-mediated circuit in Ewing's sarcoma/peripheral neuroectodermal tumor: a possible therapeutic target. Cancer Res.

[30] Scotlandi K, Benini S, Nanni P, Lollini PL, Nicoletti G, Landuzzi L, Serra M, Manara MC, Picci P, Baldini N (1998). Blockage of insulin-like growth factor-I receptor inhibits the growth of Ewing's sarcoma in athymic mice. Cancer Res.

[31] Scotlandi K, Maini C, Manara MC, Benini S, Serra M, Cerisano V, Strammiello R, Baldini N, Lollini PL, Nanni P, Nicoletti G, Picci P (2002). Effectiveness of insulin-like growth factor I receptor antisense strategy against Ewing's sarcoma cells. Cancer gene therapy.

[32] Toretsky JA, Kalebic T, Blakesley V, LeRoith D, Helman LJ (1997). The insulin-like growth factor-I receptor is required for EWS/FLI-1 transformation of fibroblasts. J Biol Chem.

[33] Mackintosh C, Madoz-Gurpide J, Ordonez JL, Osuna D, Herrero-Martin D (2010). The molecular pathogenesis of Ewing's sarcoma. Cancer biology & therapy.

[34] Manara MC, Nicoletti G, Zambelli D, Ventura S, Guerzoni C, Landuzzi L, Lollini PL, Maira SM, Garcia-Echeverria C, Mercuri M, Picci P, Scotlandi K (2010). NVP-BEZ235 as a new therapeutic option for sarcomas. Clin Cancer Res.

[35] Toomey EC, Schiffman JD, Lessnick SL (2010). Recent advances in the molecular pathogenesis of Ewing's sarcoma. Oncogene.

[36] Scotlandi K, Avnet S, Benini S, Manara MC, Serra M, Cerisano V, Perdichizzi S, Lollini PL, De Giovanni C, Landuzzi L, Picci P (2002). Expression of an IGF-I receptor dominant negative mutant induces apoptosis, inhibits tumorigenesis and enhances chemosensitivity in Ewing's sarcoma cells. Int J Cancer.

[37] Scotlandi K, Manara MC, Nicoletti G, Lollini PL, Lukas S, Benini S, Croci S, Perdichizzi S, Zambelli D, Serra M, Garcia-Echeverria C, Hofmann F, Picci P (2005). Antitumor activity of the insulin-like growth factor-I receptor kinase inhibitor NVP-AEW541 in musculoskeletal tumors. Cancer Res.

[38] Sankar S, Bell R, Stephens B, Zhuo R, Sharma S, Bearss DJ, Lessnick SL (2013). Mechanism and relevance of EWS/FLI-mediated transcriptional repression in Ewing sarcoma. Oncogene.

[39] Grunewald TG, Diebold I, Esposito I, Plehm S, Hauer K, Thiel U, da Silva-Buttkus P, Neff F, Unland R, Muller-Tidow C, Zobywalski C, Lohrig K, Lewandrowski U, Sickmann A, Prazeres da Costa O, Gorlach A (2012). STEAP1 is associated with the invasive and oxidative stress phenotype of Ewing tumors. Molecular cancer research : MCR.

[40] Surdez D, Benetkiewicz M, Perrin V, Han ZY, Pierron G, Ballet S, Lamoureux F, Redini F, Decouvelaere AV, Daudigeos-Dubus E, Geoerger B, de Pinieux G, Delattre O, Tirode F (2012). Targeting the EWSR1-FLI1 oncogene-induced protein kinase PKC-beta abolishes ewing sarcoma growth. Cancer Res.

[41] Tirado OM, Mateo-Lozano S, Villar J, Dettin LE, Llort A, Gallego S, Ban J, Kovar H, Notario V (2006). Caveolin-1 (CAV1) is a target of EWS/FLI-1 and a key determinant of the oncogenic phenotype and tumorigenicity of Ewing's sarcoma cells. Cancer Res.

[42] Olmos D, Postel-Vinay S, Molife LR, Okuno SH, Schuetze SM, Paccagnella ML, Batzel GN, Yin D, Pritchard-Jones K, Judson I, Worden FP, Gualberto A, Scurr M, de Bono JS, Haluska P (2010). Safety, pharmacokinetics, and preliminary activity of the anti-IGF-1R antibody figitumumab (CP-751,871) in patients with sarcoma and Ewing's sarcoma: a phase 1 expansion cohort study. Lancet Oncol.

[43] Bond M, Bernstein ML, Pappo A, Schultz KR, Krailo M, Blaney SM, Adamson PC (2008). A phase II study of imatinib mesylate in children with refractory or relapsed solid tumors: a Children's Oncology Group study. Pediatr Blood Cancer.

[44] Gualberto A, Pollak M (2009). Emerging role of insulin-like growth factor receptor inhibitors in oncology: early clinical trial results and future directions. Oncogene.

[45] Tolcher AW, Sarantopoulos J, Patnaik A, Papadopoulos K, Lin CC, Rodon J, Murphy B, Roth B, McCaffery I, Gorski KS, Kaiser B, Zhu M, Deng H, Friberg G, Puzanov I (2009). Phase I, pharmacokinetic, and pharmacodynamic study of AMG 479, a fully human monoclonal antibody to insulin-like growth factor receptor 1. J Clin Oncol.

[46] Rikhof B, de Jong S, Suurmeijer AJ, Meijer C, van der Graaf WT (2009). The insulin-like growth factor system and sarcomas. J Pathol.

[47] Scotlandi K, Picci P (2008). Targeting insulin-like growth factor 1 receptor in sarcomas. Curr Opin Oncol.

[48] Toretsky JA, Steinberg SM, Thakar M, Counts D, Pironis B, Parente C, Eskenazi A, Helman L, Wexler LH (2001). Insulin-like growth factor type 1 (IGF-1) and IGF binding protein-3 in patients with Ewing sarcoma family of tumors. Cancer.

[49] Moller E, Mandahl N, Iliszko M, Mertens F, Panagopoulos I (2009). Bidirectionality and transcriptional activity of the EWSR1 promoter region. Oncol Rep.

[50] Mateo-Lozano S, Tirado OM, Notario V (2003). Rapamycin induces the fusion-type independent downregulation of the EWS/FLI-1 proteins and inhibits Ewing's sarcoma cell proliferation. Oncogene.

[51] Baliko F, Bright T, Poon R, Cohen B, Egan SE, Alman BA (2007). Inhibition of notch signaling induces neural differentiation in Ewing sarcoma. Am J Pathol.

[52] Hingorani P, Dickman P, Garcia-Filion P, White-Collins A, Kolb EA, Azorsa DO (2013). BIRC5 expression is a poor prognostic marker in Ewing sarcoma. Pediatr Blood Cancer.

[53] Greve B, Sheikh-Mounessi F, Kemper B, Ernst I, Gotte M, Eich HT (2012). Survivin, a target to modulate the radiosensitivity of Ewing's sarcoma. Strahlenther Onkol.

[54] Zhang Y, Liao M, Dufau ML (2006). Phosphatidylinositol 3-kinase/protein kinase Czeta-induced phosphorylation of Sp1 and p107 repressor release have a critical role in histone deacetylase inhibitor-mediated derepression [corrected] of transcription of the luteinizing hormone receptor gene. Mol Cell Biol.

[55] Grohar PJ, Woldemichael GM, Griffin LB, Mendoza A, Chen QR, Yeung C, Currier DG, Davis S, Khanna C, Khan J, McMahon JB, Helman LJ (2011). Identification of an inhibitor of the EWS-FLI1 oncogenic transcription factor by high-throughput screening. J Natl Cancer Inst.

[56] Mireuta M, Darnel A, Pollak M (2010). IGFBP-2 expression in MCF-7 cells is regulated by the PI3K/AKT/mTOR pathway through Sp1-induced increase in transcription. Growth Factors.

[57] Kurzrock R, Patnaik A, Aisner J, Warren T, Leong S, Benjamin R, Eckhardt SG, Eid JE, Greig G, Habben K, McCarthy CD, Gore L (2010). A phase I study of weekly R1507, a human monoclonal antibody insulin-like growth factor-I receptor antagonist, in patients with advanced solid tumors. Clin Cancer Res.

[58] Huang HJ, Angelo LS, Rodon J, Sun M, Kuenkele KP, Parsons HA, Trent JC, Kurzrock R (2011). R1507, an anti-insulin-like growth factor-1 receptor (IGF-1R) antibody, and EWS/FLI-1 siRNA in Ewing's sarcoma: convergence at the IGF/IGFR/Akt axis. PLoS One.

[59] Barber-Rotenberg JS, Selvanathan SP, Kong Y, Erkizan HV, Snyder TM, Hong SP, Kobs CL, South NL, Summer S, Monroe PJ, Chruszcz M, Dobrev V, Tosso PN, Scher LJ, Minor W, Brown ML (2012). Single enantiomer of YK-4–279 demonstrates specificity in targeting the oncogene EWS-FLI1. Oncotarget.

[60] Nelson JD, Denisenko O, Bomsztyk K (2006). Protocol for the fast chromatin immunoprecipitation (ChIP) method. Nat Protoc.

