# Supplementary material for: PI3K/AKT signaling modulates transcriptional expression of EWS/FLI1 through specificity protein 1
Source: Oncotarget. 2015 Aug 22;6(30):28895–910. doi: 10.18632/oncotarget.5000 (PMC4745699; doi:10.18632/oncotarget.5000)
Supplement: Supplementary file 1 [file oncotarget-06-28895-s001.pdf]

SUPPLEMENTARY FIGURES AND TABLES

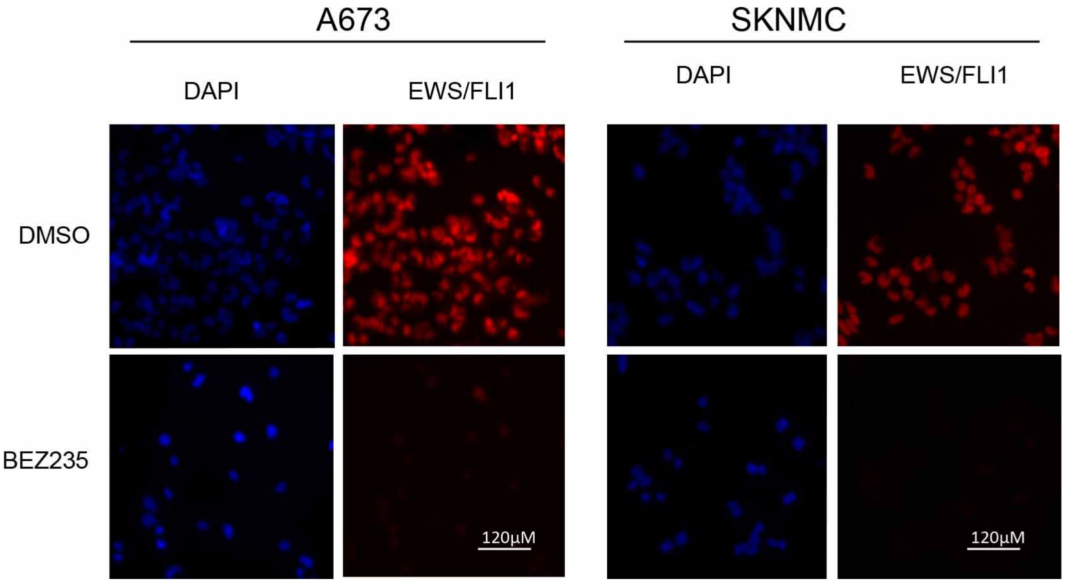

Supplementary Figure S1: ***BEZ235 treatment affects EWS/FLI1 level.*** Representative experiment ($n = 3$) of immunofluorescence assessment of EWS/FLI1 after BEZ235 treatment for 24 hrs in A673 and in SKNMC cells.

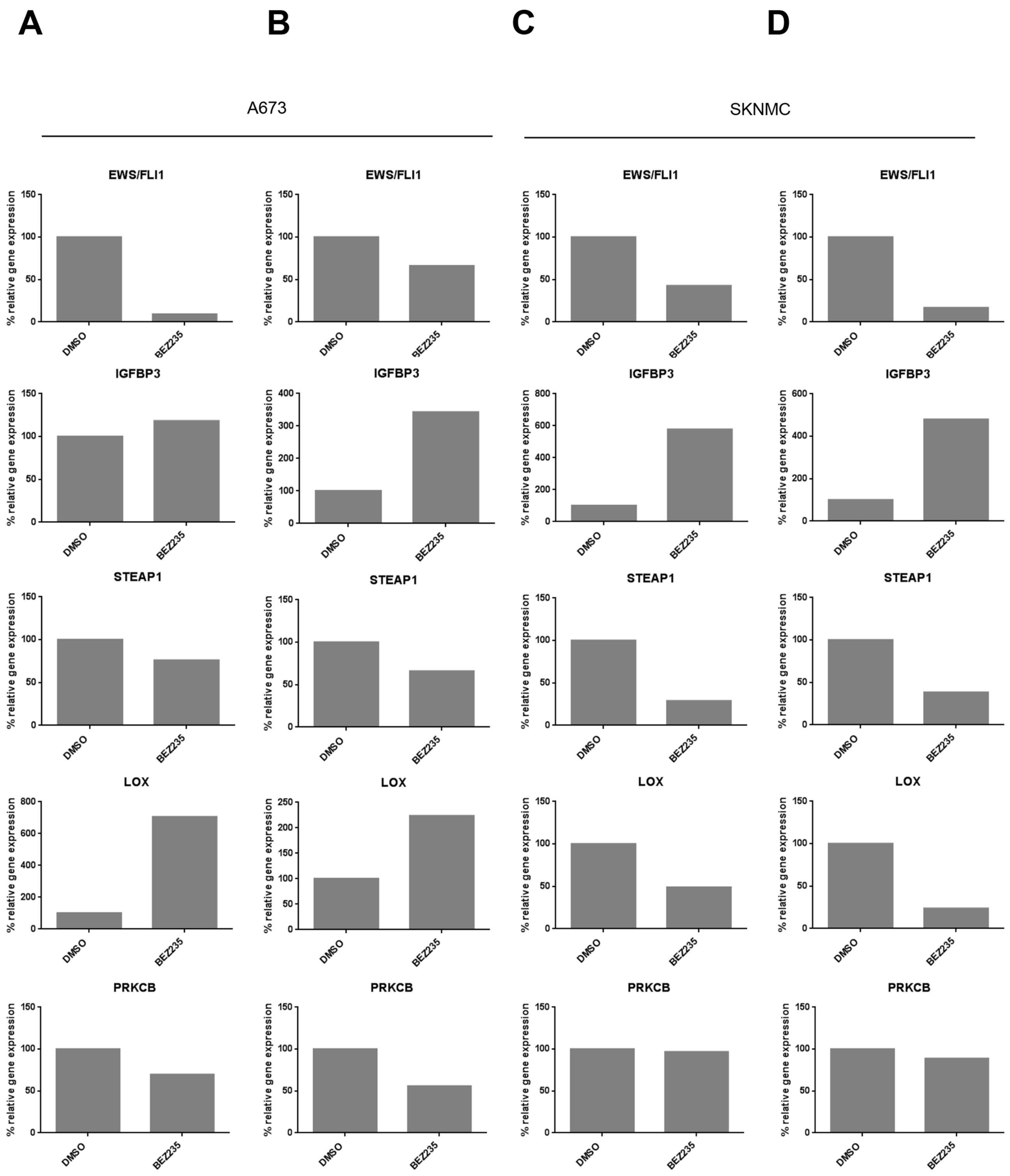

Supplementary Figure S2: ***BEZ235 treatment affects EWS/FLI1 gene expression and its target genes.*** Gene expression level in percentage of EWS/FLI1 and its repressed (LOX and IGFBP3) and activated (PRKCB and STEAP1) target genes after BEZ235 treatment in A673 **A, B.** and in SKNMC cells **C, D.** for 24 (A,C) and 48 hrs (B,D). Shown is a representative experiment (*n* = 3).

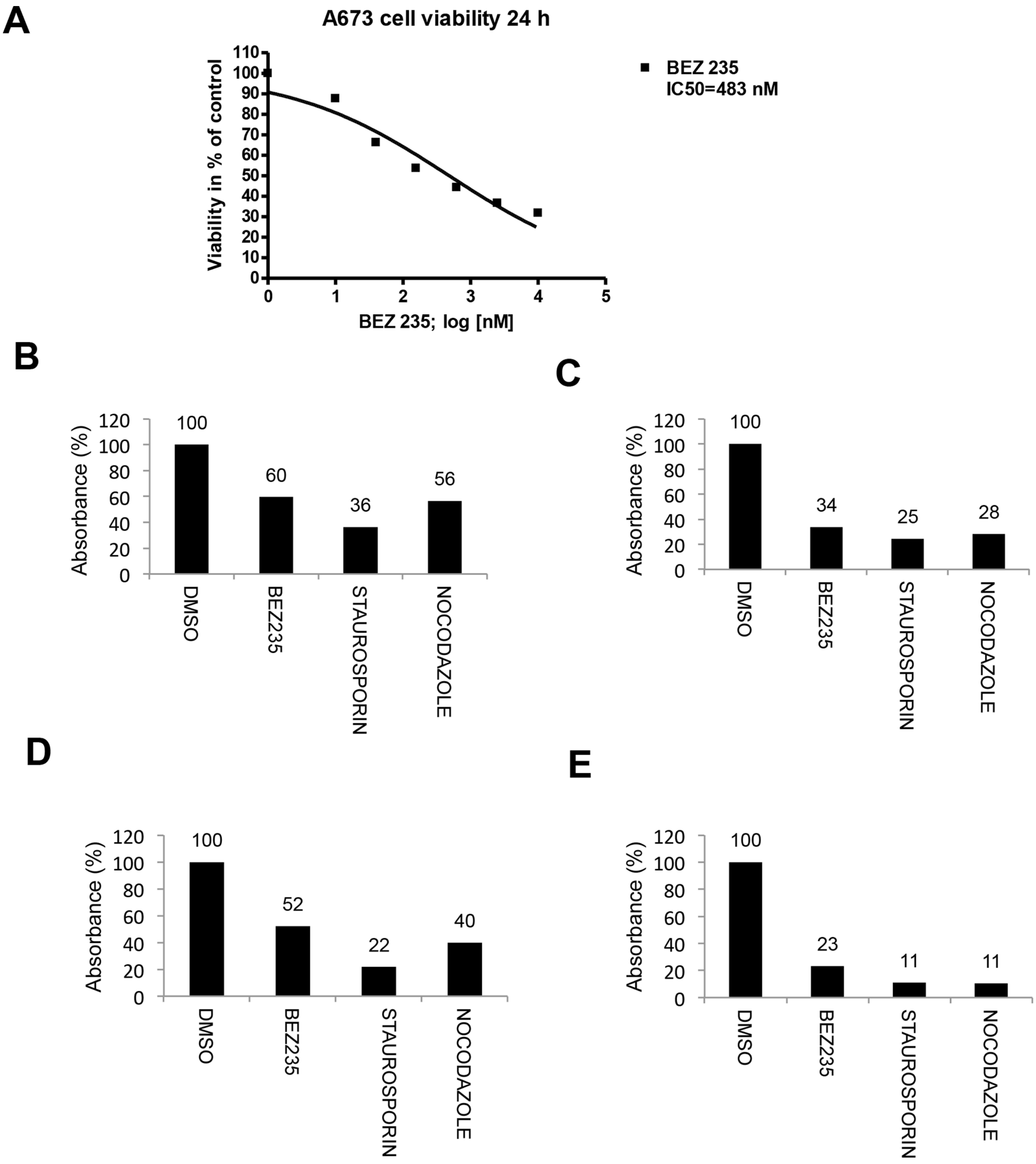

Supplementary Figure S3: A. A673 cells viability after 24 hrs treatment with titrating concentration of BEZ235. Absorbance in percentage compared to DMSO treated control after drug treatment for 24 and 48 hrs in A673 **B.** and **C.** and SKNMC cells **D.** and **E.** Bars represent mean values of 8 biological replicates analysed in three technical replicates each ($n = 3$; SE < 0.01).

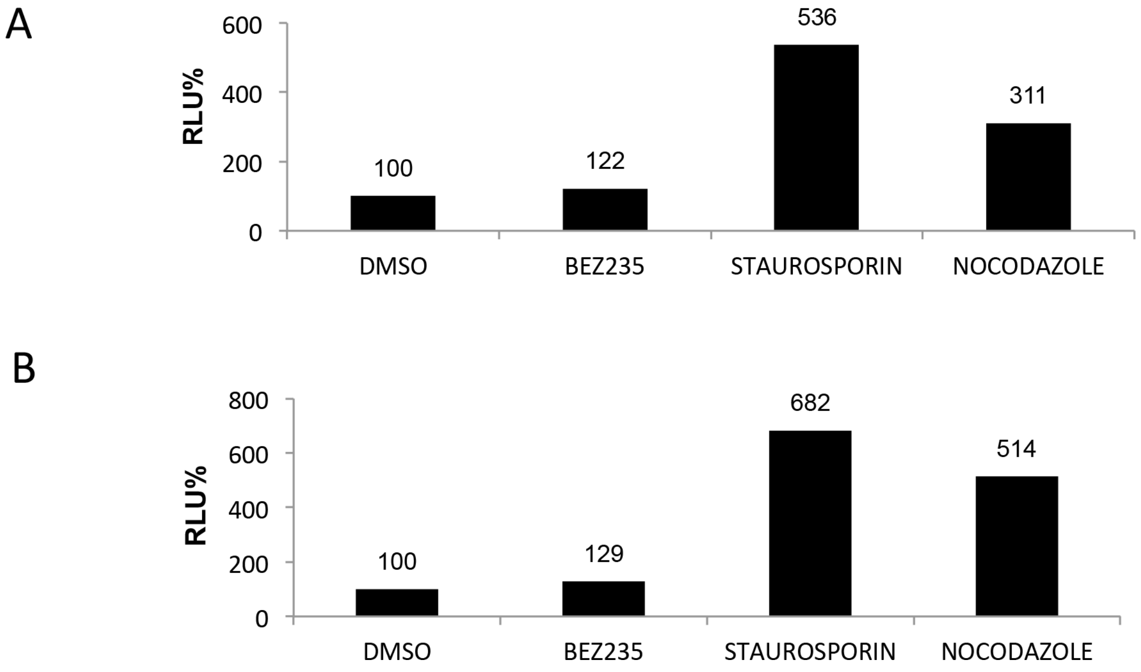

Supplementary Figure S4: ***CASPASE 3/7 ACTIVITY.*** Caspase 3/7 activity measured after 24 hrs treatment with 500 nM BEZ235, 1 µM Staurosporin and 100 nM Nocodazole in A673 **A.** and SKNMC cells **B.** Bars represent mean values expressed as relative light unit (RLU) in percentage of DMSO treated control of 6 biological replicate analysed in three technical replicates each (n = 3; SE < 0.01).

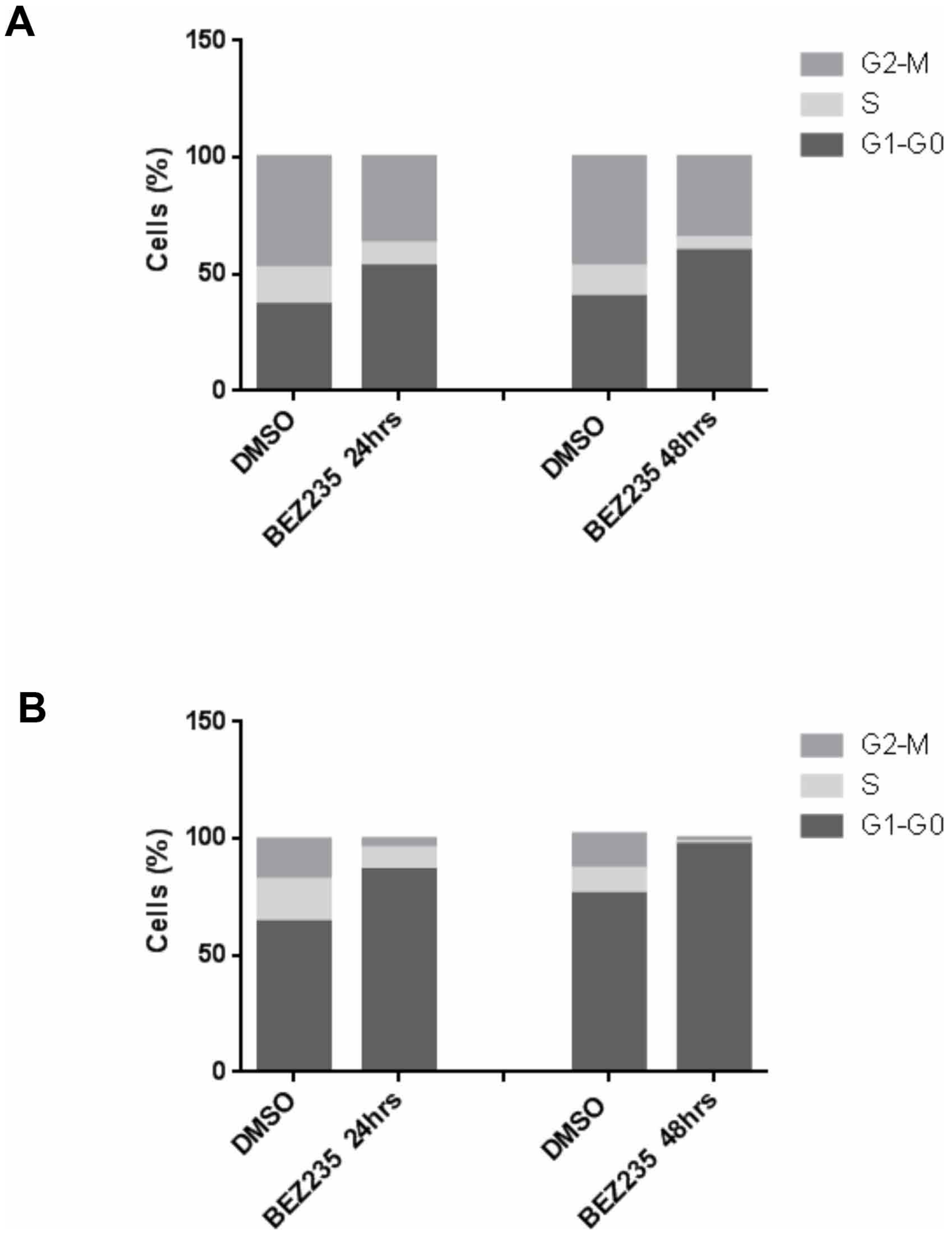

Supplementary Figure S5: ***Cell cycle progression upon BEZ235 treatment.*** Cell cycle analysis after 24 and 48 hrs treatment with 500 nM BEZ235 compared to DMSO treated control in A673 **A.** and SKNMC **B.** Shown is a representative experiment (*n* = 3).

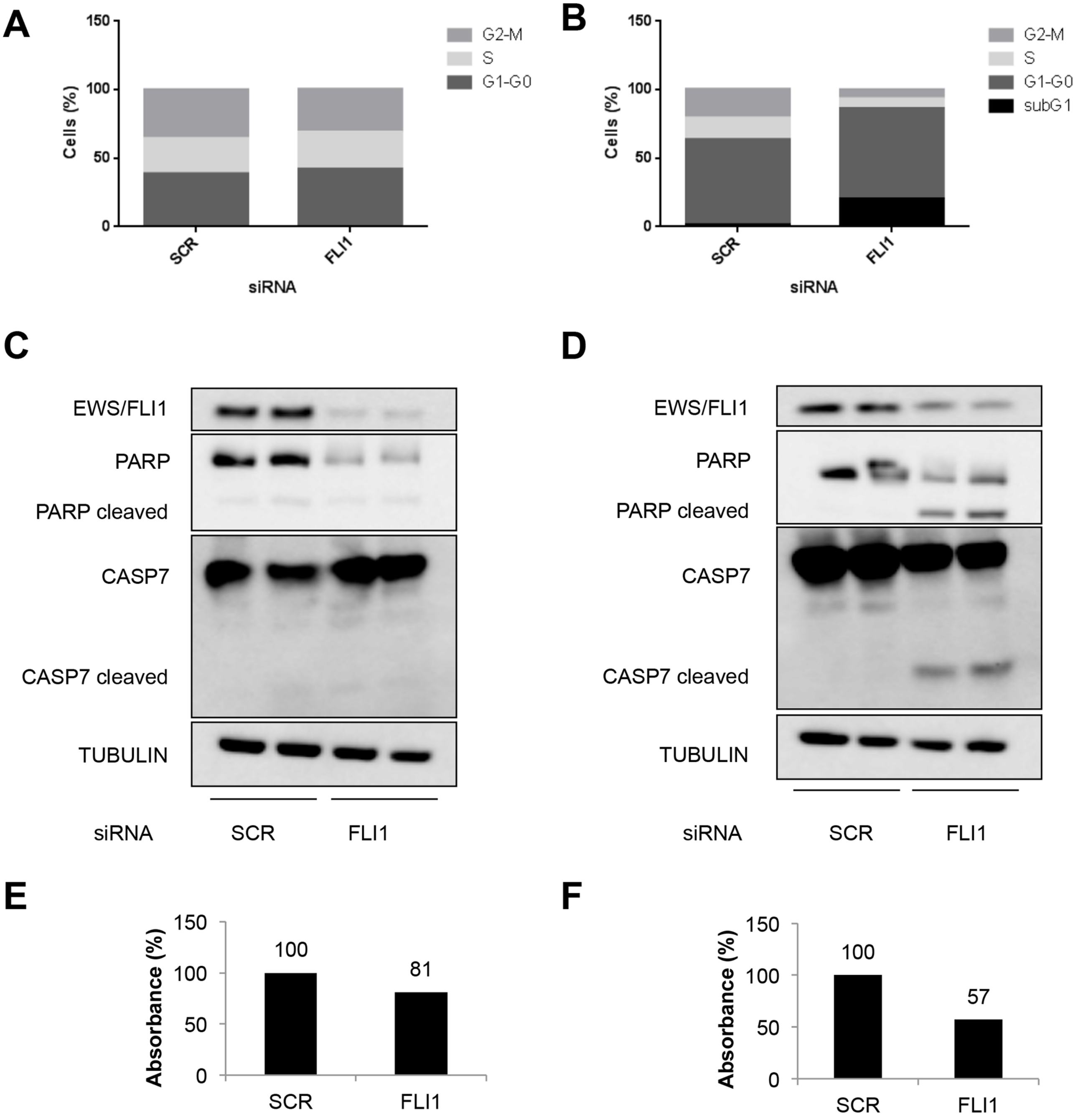

Supplementary Figure S6: ***Cell cycle progression after silencing of EWS/FLI1.*** Cell cycle analysis upon EWS/FLI1 depletion for 48 hrs in A673 **A.** and SKNMC cells **B.** Protein level measured by western blot of EWS/FLI1, PARP, CASP7 and TUBULIN in A673 **C.** and SKNMC cells **D.** after silencing of EWS/FLI1 in biological duplicates. **E.** Absorbance in percentage of viable cells upon depletion of EWS/FLI1 in A673 and SKNMC cells **F.** compared to Scr control . Shown are representative experiments (n = 3).

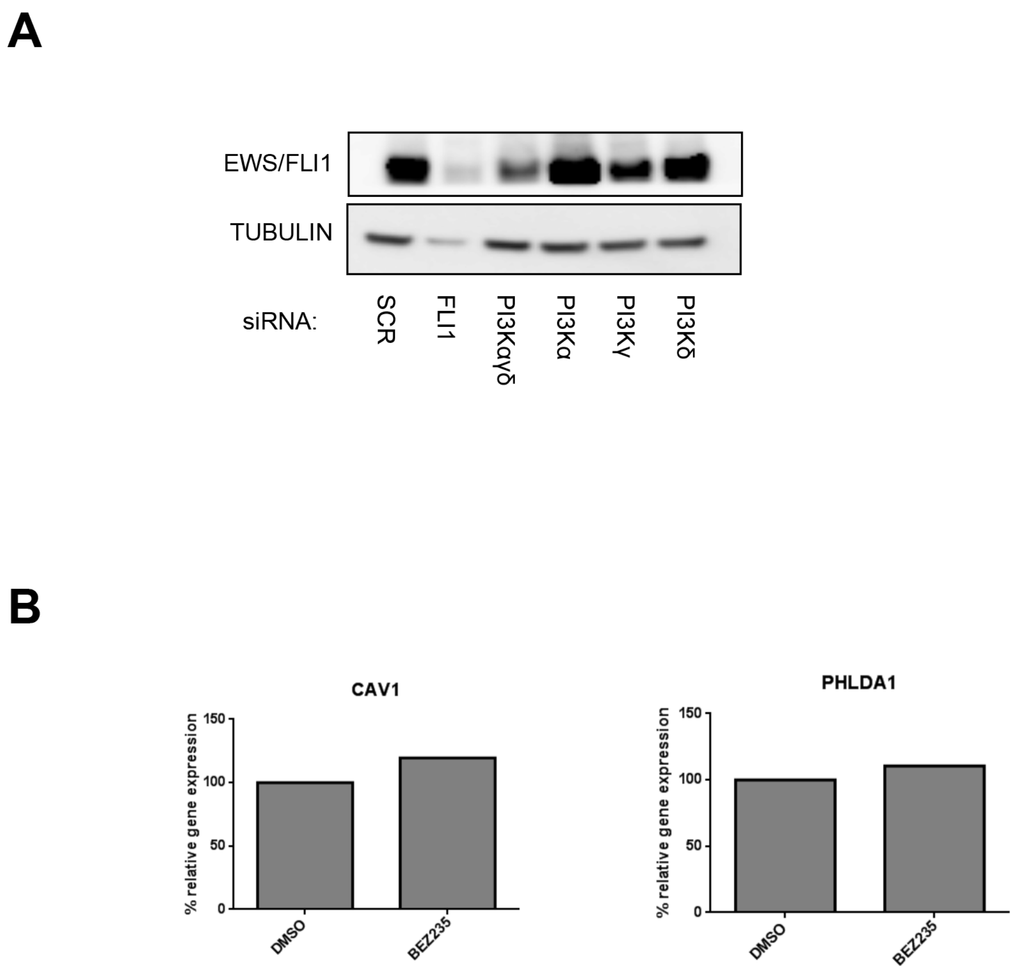

Supplementary Figure S7: ***Effects of PI3K pathway inhibition on EWS/FLI1 and ist target genes.*** **A.** siRNA mediated knockdown of PI3K components for 48 hrs in order to determine EWS/FLI1 level in SKNMC cells. **B.** mRNA level of EWS/FLI1 target genes expressed endogenuously in PC3 cells after BEZ235 treatment for 24 hrs. Shown are representative experiments (*n* = 3).

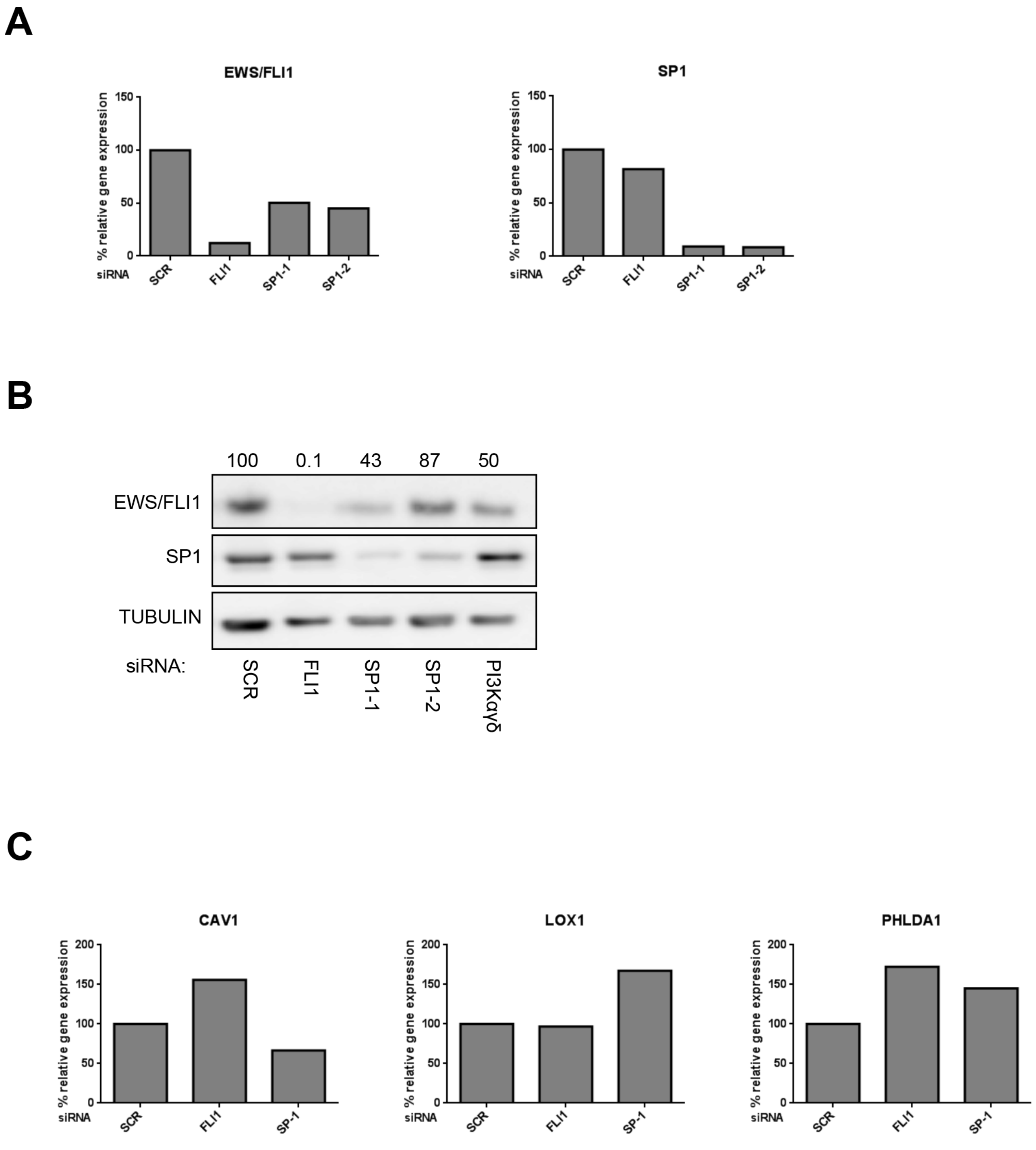

Supplementary Figure S8: ***Effects of SP1 silencing on EWS/FLI1 level and ist target genes.*** **A.** siRNA mediated knockdown of SP1 for 48 hrs in order to determine mRNA expression of EWS/FLI1 , and SP1 (via qRT-PCR) in SKNMC cells. **B.** Protein level assesment of EWS/FLI1 via western blot after sliencing of SP1 or PI3Kαγδ in SKNMC cells. **C.** mRNA level of EWS/FLI1 target genes expressed endogenuously in PC3 cells after silencing of SP1 for 48 hrs. Shown are representative experiments (*n* = 3).

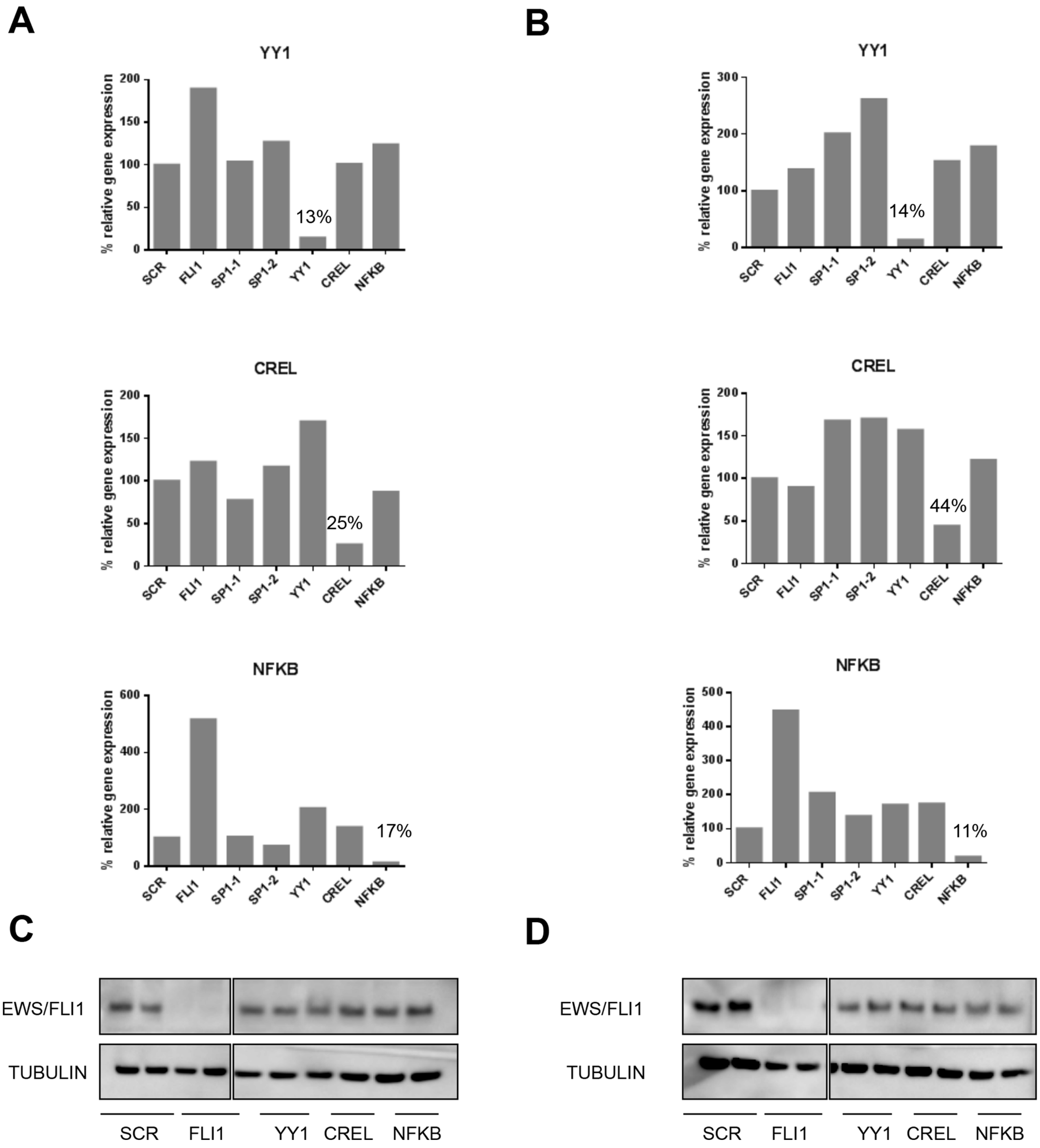

Supplementary Figure S9: ***Validation of the knock down of transcription factors candidates for the Del23 region and its effect on EWS/FLI1.*** Gene expression data after reverse knock down candidates for Del23 region in SKNMC **A.** and A673 **B.** EWS/FLI1 protein level after silencing for 48 hrs of the candidate transcription factors by siRNA in SKNMC cells **C.** and A673 **D.** Shown are representative experiments (*n* = 3).

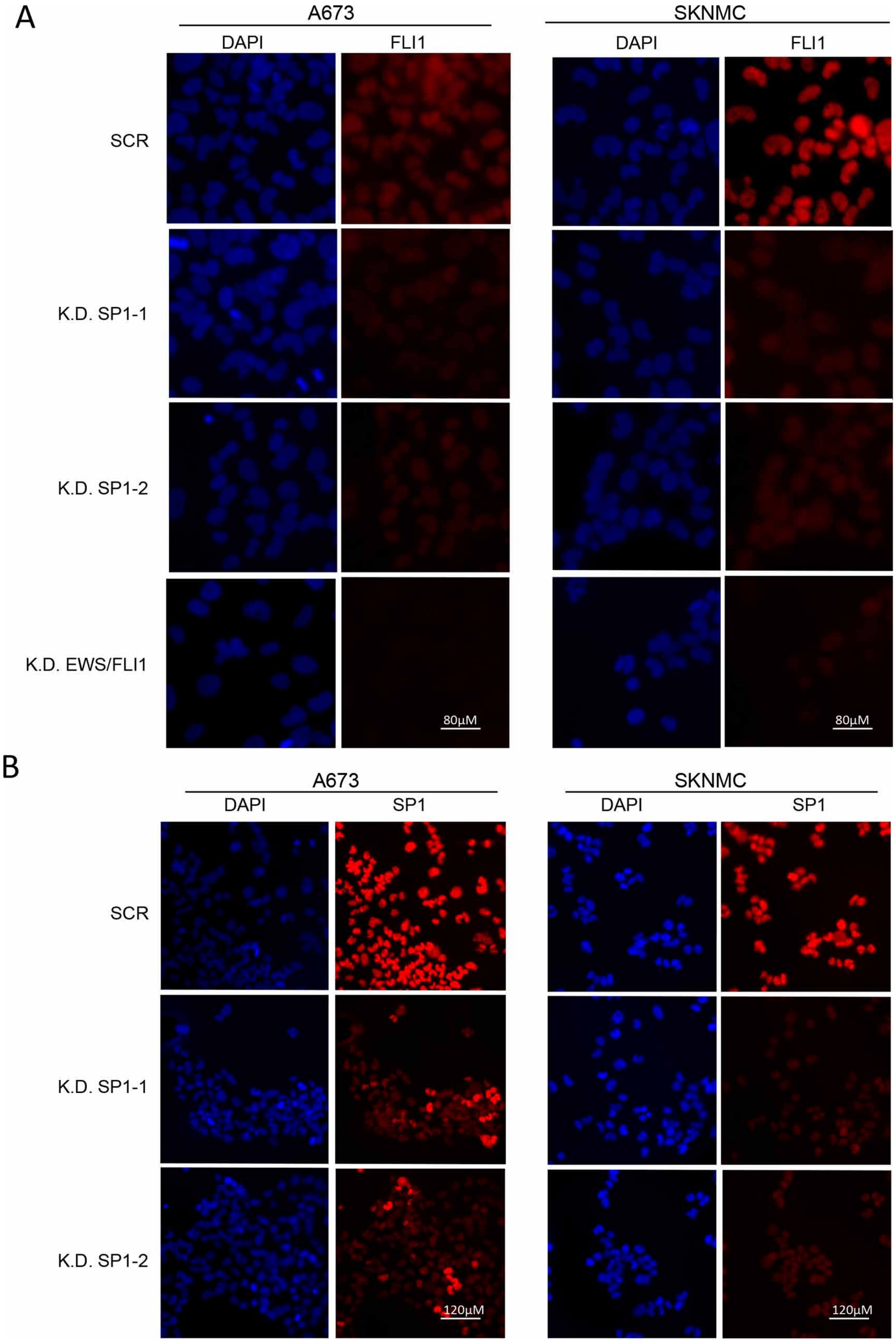

Supplementary Figure S10: ***SP1 knock down affects EWS/FLI1 level.*** Immunofluorescence assessment of EWS/FLI1 **A.** and SP1 **B.** after reverse knock down of SP1 in A673 and SKNMC cells. Exposure times were kept constant for all samples.

Supplementary Table S1: List of targeted inhibitors used in the screening

No.	Name	Target
1	[Ala92]-p16 (84–103)	Cdk inhibitor
2	17-AAG	Hsp90 inhibitor
3	2-Deoxy-D-glucose	Glycolysis inhibitor
4	3-MA	Vps34 inhibitor (class III PI3K)
5	A 769662	AMPK activator
6	ABT-102	TRVP1 antagonist
7	ABT-737	BCL-2, BCL-xl inhibitor
8	AEG 3482	JNK inhibitor
9	AG 013736 - Axitinib	VEGFR inhibitor
10	AMD3100	CXCR4 inhibitor
11	AMN 107 (Nilotinib)	BCR-ABL inhibitor
12	Apratastat	TACE/MMP inhibitor
13	AS 252424	PI3K p110 gamma inhibitor
14	AT9283	Aurora kinases Inhibitor
15	Atazanivir	Protease inhibitor
16	AZD 2281 - Olaparib	PARP inhibitor
17	AZD 7762	CHK inhibitor
18	AZD1152-HQPA	Aurora B inhibitor
19	BACE 1 inhibitor	Beta Secretase inhibitor
20	Bax inhibitor peptide P5	Bcl-2 Protein Family Inhibitor
21	Bax inhibitor peptide V5	Bcl-2 Protein Family Inhibitor
22	Bax inhibitor peptide,negative control	Bcl-2 Protein Family Inhibitor
23	BAY 61–3606	Syk inhibitor
24	BI 2536	PLK-1 inhibitor
25	BMS 189961	RAR gamma agonist
26	BMS 270394	RAR gamma agonist
27	BMS-345541	IκB inhibitor
28	Bosutinib (SKI 606)	BCR-ABL/SRC inhibitor
29	Butabindide	TPPII inhibitor
30	BX 795	PDK1/TBK1 inhibitor
31	BX 912	PDK1 inhibitor
32	BZ - Gamma Secretase inhibitor BZ	Gamma Secretase inhibitor
33	Cediranib	VEGFR inhibitor
34	CH 55	RAR alpha/beta agonist

(*Continued*)

No.	Name	Target
35	Chir98014	GSK-3 inhibitor
36	CI-1033 - Canertinib	EGFR inhibitor
37	Combretastatin-A4 (CA-4)	Inhibitor of tubulin polymerization
38	Compound C	AMPK inhibitor
39	CP 690550	JAK3 inhibitor
40	CT 99021 - CHIR 99021	GSK-3 inhibitor
41	Cyclopamine	Hedgehog Pathway Inhibitor
42	Cyclosporine	Immunosupressant
43	DAPT	Gamma Secretase inhibitor
44	Dasatinib	BCR-ABL/SRC inhibitor
45	DBZ	Gamma Secretase inhibitor
46	Deguelin	Anticancer agent
47	DM 3189	BMP inhibitor
48	Doramapimod	p38 MAPK inhibitor
49	DR 2313	PARP inhibitor
50	FK 866	NAPRT1 inhibitor, anti-cancer agent
51	GDC 0879	B-Raf inhibitor
52	GDC 0941	PI3K inhibitor
53	GDC-0449	Hedgehog Pathway Inhibitor
54	Gefitinib	EGFR inhibitor
55	GSK 269962A	ROCK1 inhibitor
56	GW 441756	TrkA inhibitor
57	GW 786034	VEGFR/KIT/PDGFR inhibitor
58	GW 843682X	PLK inhibitor
59	Honokiol	pAkt, scr, p44/42 MAPK
60	HU-308	CB2 agonist
61	IGC-001	Beta-Catenin inhibitor
62	Imatinib	BCR-ABL inhibitor
63	JAK inhibitor I	JAKs inhibitor
64	JIP-1 (153–163)	JNK Inhibitor
65	JWH 018	CB2 agonist
66	JWH 073	CB1/2 agonist
67	JWH 133	CB2 agonist
68	Ko 143	BCRP inhibitor
69	KU-55933	ATM inhibitor
70	L-685,485	Gamma Secretase inhibitor

(*Continued*)

No.	Name	Target
71	L-aminoadipic acid	Glutamine synthase inhibitor
72	Lamotrigine	Glutamate antagonist
73	Lapatinib	EGFR/ErbB-2 inhibitor
74	LE-135	RAR beta antagonist
75	LY 2157299	TGF beta receptor inhibitor
76	LY 294002	PI3K inhibitor
77	Masitinib mesylate	KIT/PDGFR inhibitor
78	MK 1775	Wee1 inhibitor
79	MK-2206	Akt inhibitor
80	MLN8237	Aurora kinase inhibitor
81	Myoseverin	Microtubule inhibitor
82	Na-Butyrate	HDAC inhibitor
83	NEC-1	Necroptosis/RIPK inhibitor
84	NF-kB Inhibitor	NF-kB Inhibitor
85	Nocodazole	Cell cycle G2/M inhibitor
86	NSC 348884	Nucleophosmin inhibitor
87	NSC 625987	CDK4 inhibitor
88	NU 1025	PARP inhibitor
89	NU 7441	DNA-PK inhibitor
90	NVP-AEW514	IGF-1R inhibitor
91	NVP-AUY922	HSP90 inhibitor
92	NVP-BAG956	PI3K/PDK1 inhibitor
93	NVP-BEZ235	PI3K/mTOC inhibitor
94	NVP-BGJ398	FGF-R inhibitor
95	NVP-BKM120	PI3K, not mTOC inhibitor
96	NVP-BSK805	JAK2 inhibitor
97	NVP-TAE684	ALK inhibitor
98	NVP-TKI258	FGF-R inhibitor
99	Obatoclax	Bcl-2 inhibitor
100	OSI 774 - Erlotininb	EGFR inhibitor
101	OSI-027	mTORC1/2 inhibitor
102	Palmitoylethanolamide	Endocannabinoid
103	PD 0325901	MEK inhibitor
104	PD 166793	MMP inhibitor
105	PD 169316	p38 MAPK inhibitor
106	PD 180970	Src kinase inhibitor
107	PD 184352	MEK 1 inhibitor

(*Continued*)

No.	Name	Target
108	PD 98059	MEK inhibitor
109	PD150606	Calpain inhibitor
110	PF-00356231	MMP-12 inhibitor
111	PHA-739358(Danusertib)	Aurora kinases,Bcr-Abl and FGFR inhibitor
112	PI 103	Class I PI3K inhibitor
113	piceatannol	Syk inhibitor
114	PIK 75	PI3K p110 alpha inhibitor
115	PIK 90	PI3K p110 alpha inhibitor
116	pimecrolimus	Calcineurin inhibitor
117	PLX 4720	B-Raf inhibitor
118	PP2	Src inhibitor
119	PP242	mTORC1/2 inhibitor
120	PU-H71	HSP90 inhibitor
121	Roscovitine/Celiciblib	Cdk inhibitor
122	Ruboxistaurin (LY333531)	PKC beta inhibitor
123	S31–201	Stat3 Inhibitor
124	Saracatinib	Src and Abl inhibitor
125	SB 202190	p38 MAPK inhibitor
126	SB 203580	p38 MAPK inhibitor
127	SB 216763	GSK-3 inhibitor
128	SB 431542	TGF beta receptor inhibitor
129	Scriptaid	HDAC inhibitor
130	SD 169	p38 MAPK inhibitor
131	SD 208	TGF-betaR receptor 1 inhibitor
132	SL 327	MEK1/2 inhibitor
133	SNS-314	Aurora kinase inhibitor
134	Sorafenib (BAY 43–9006)	Raf/Mek/Erk inhibitor
135	Stobadine	Antioxidant
136	SU 6656	Src kinase inhibitor
137	SU11274	c-Met inhibitor
138	Sunitinib - SU 11248	Multiple RTK inhibitor
139	Tacrolimus	Calcineurin inhibitor
140	Tandutinib	FLT3 inhibitor
141	TG101348	JAK2 (Flt3) inhibitor
142	TGX 221	PI3K p110 beta inhibitor
143	Tiplaxtinin	PAI-1 inhibitor
144	TW-37	Bcl-2 protein family inhibitor

(*Continued*)

No.	Name	Target
145	Tyrphostin AG 490	JAK2 inhibitor
146	U 73122	Phospholipase C inhibitor
147	Vandetanib	VEGFR/EGFR Inhibitor
148	Velcade	Proteasome inhibitor
149	Vorinostat/SAHA	HDAC inhibitor
150	VPA	HDAC inhibitor
151	xav-939	Wnt/beta-catenin signal transd. inhibitor
152	XL228	Multiple Tyr-kinase inhib. (IGF1-R, Bcr-Abl)
153	Y-27632	p160 ROCK inhibitor

Supplementary Table S2: Deletion constructs of the 2.3kb EWS/FLI1 promoter construct

Name of the construct	Position of the deletion relative to the transcription initiation site
Del1	−214/−203
Del2	−202/−191
Del3	−190/−179
Del4	−178/−167
Del5	−166/−155
Del6	−154/−143
Del7	−142/−131
Del8	−130/−119
Del9	−118/−107
Del10	−106/−95
Del11	−94/−83
Del12	−82/−71
Del13	−70/−59
Del14	−58/−47
Del15	−46/−35
Del16	−34/−23
Del17	−22/−11
Del18	−10/+2
Del19	+3/+14
Del20	+15/+26
Del21	+27/+40
Del22	+41/+54
Del23	+55/+68
Del24	+69/+80
